# Cold storage of human precision-cut lung slices in TiProtec preserves cellular composition and transcriptional responses and enables on-demand mechanistic studies

**DOI:** 10.1186/s12931-025-03132-w

**Published:** 2025-02-17

**Authors:** M. Camila Melo-Narvaez, Fee Gölitz, Eshita Jain, Janine Gote-Schniering, Mircea Gabriel Stoleriu, Wilhelm Bertrams, Bernd Schmeck, Ali Önder Yildirim, Ursula Rauen, Timo Wille, Mareike Lehmann

**Affiliations:** 1 Comprehensive Pneumology Center with the CPC-M bioArchive and Institute of Lung Health and Immunity, Helmholtz Center Munich, German Center for Lung Research (DZL), Munich, Germany; 2 Institute for Lung Research, Philipps-University Marburg, German Center for Lung Research (DZL), Marburg, Germany; 3 Bundeswehr Institute of Pharmacology and Toxicology, Munich, Germany; 4 Walther-Straub-Institute of Pharmacology and Toxicology, Ludwig-Maximilians-University, Munich, Germany; 5 Department of Rheumatology and Immunology, Department of Pulmonary Medicine, Allergology and Clinical Immunology, Inselspital, Bern University Hospital, University of Bern, Bern, Switzerland; 6 Lung Precision Medicine (LPM), Department for BioMedical Research (DBMR), University of Bern, Bern, Switzerland; 7 Division for Thoracic Surgery Munich, Ludwig-Maximilians-University of Munich (LMU) and Asklepios Lung Clinic Munich-Gauting, Gauting, Germany; 8 Core Facility Flow Cytometry - Bacterial Vesicles, Philipps-University Marburg, Marburg, Germany; 9 Department of Medicine, Pulmonary and Critical Care Medicine, University Medical Center Marburg, Philipps-University Marburg, German Center for Lung Research (DZL), Marburg, Germany; 10 Center for Synthetic Microbiology (Synmikro), Philipps-University Marburg, Marburg, Germany; 11 Member of the German Center of Infectious Disease Research, Marburg, Germany; 12 Institute of Experimental Pneumology (IEP), Ludwig-Maximilians University of Munich (LMU), Munich, Germany; 13 Institute of Physiological Chemistry, University Hospital Essen, Essen, Germany; 14 Department of CBRN Medical Defense, Bundeswehr Medical Academy, Munich, Germany; 15 Institute for Lung Health (ILH), German Center for Lung Research (DZL), Giessen, Germany

**Keywords:** Human precision-cut lung slices (hPCLS), 3R, Long-term cold storage, Tissue preservation, Fibrosis, Human lung models, TiProtec

## Abstract

**Background:**

Human precision-cut lung slices (hPCLS) are a unique platform for functional, mechanistic, and drug discovery studies in the field of respiratory research. However, tissue availability, generation, and cultivation time represent important challenges for their usage. Therefore, the present study evaluated the efficacy of a specifically designed tissue preservation solution, TiProtec, complete or in absence (-) of iron chelators, for long-term cold storage of hPCLS.

**Methods:**

hPCLS were generated from peritumor control tissues and stored in DMEM/F-12, TiProtec, or TiProtec (-) for up to 28 days. Viability, metabolic activity, and tissue structure were determined. Moreover, bulk-RNA sequencing was used to study transcriptional changes, regulated signaling pathways, and cellular composition after cold storage. Induction of cold storage-associated senescence was determined by transcriptomics and immunofluorescence (IF). Finally, cold-stored hPCLS were exposed to a fibrotic cocktail and early fibrotic changes were assessed by RT-qPCR and IF.

**Results:**

Here, we found that TiProtec preserves the viability, metabolic activity, transcriptional profile, as well as cellular composition of hPCLS for up to 14 days. Cold storage did not significantly induce cellular senescence in hPCLS. Moreover, TiProtec downregulated pathways associated with cell death, inflammation, and hypoxia while activating pathways protective against oxidative stress. Cold-stored hPCLS remained responsive to fibrotic stimuli and upregulated extracellular matrix-related genes such as fibronectin and collagen 1 as well as alpha-smooth muscle actin, a marker for myofibroblasts.

**Conclusions:**

Optimized long-term cold storage of hPCLS preserves their viability, metabolic activity, transcriptional profile, and cellular composition for up to 14 days, specifically in TiProtec. Finally, our study demonstrated that cold-stored hPCLS can be used for on-demand mechanistic studies relevant for respiratory research.

**Graphical Abstract:**

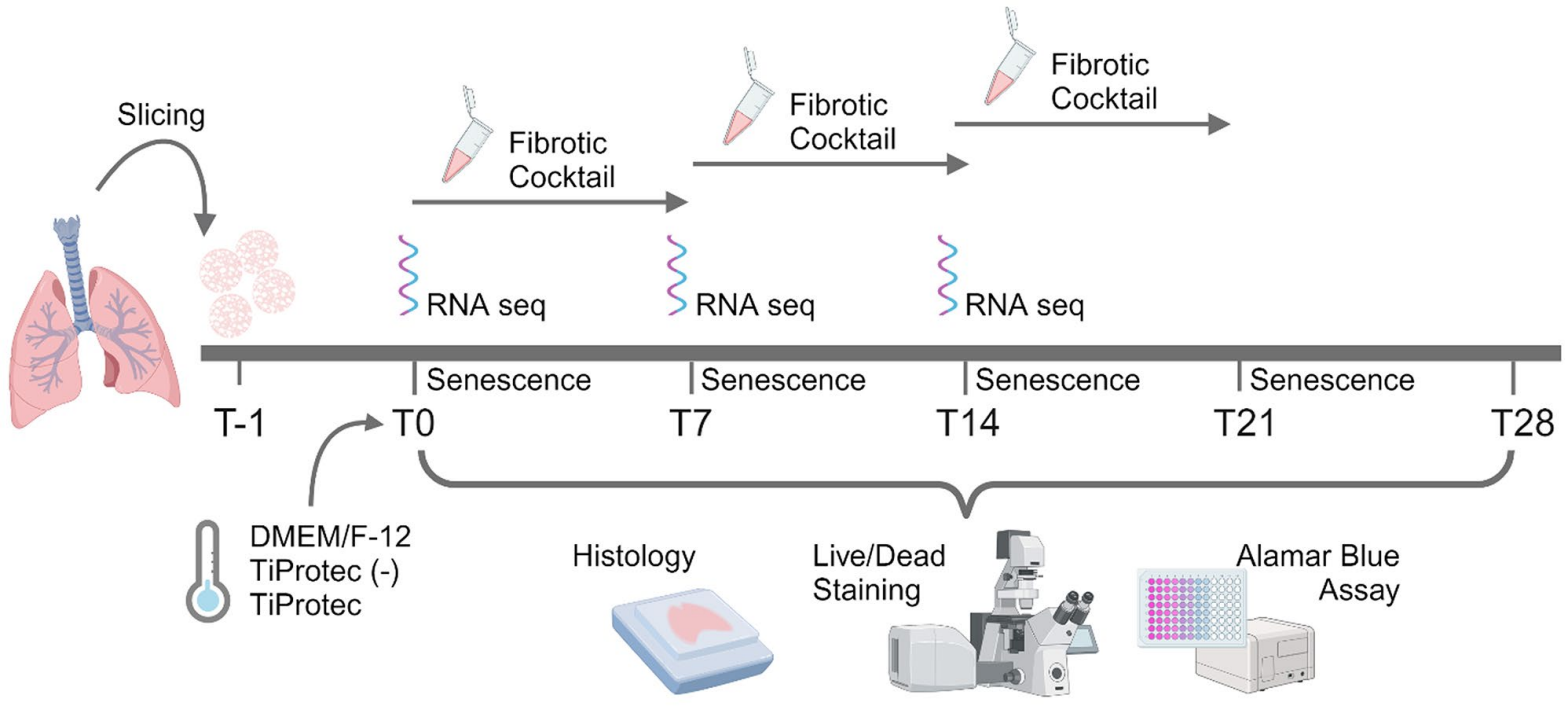

**Supplementary Information:**

The online version contains supplementary material available at 10.1186/s12931-025-03132-w.

## Background

Chronic lung diseases represent a significant health burden and are among the leading causes of morbidity and mortality worldwide [1]. Several models have been used in the past to study respiratory diseases and human derived ex vivo models such as Precision-Cut Lung Slices (PCLS) are versatile models for studying the underlying mechanisms [2–4], evaluating impact of novel therapeutics [5–7], and examining (toxic) environmental impacts on lungs [8–10]. Their importance in basic research and drug development lies in their ability to maintain the intact lung architecture, since resident cell types including epithelial, mesenchymal, and resident innate and adaptive immune cells as well as the extracellular matrix (ECM) are maintained in their native three-dimensional (3D) structure (11–13). Therefore, PCLS can be applied to study various endpoints, including viability, metabolic activity as well as ECM changes in response to a fibrotic stimulation [4, 14–16]. Notably, advanced microscopy technologies can be applied to study longitudinal and functional responses of PCLS [17–22] and recently, single-cell-RNA sequencing datasets obtained from PCLS have provided new insights into the mechanisms of the progression of fibrosis and pointed to possible new therapeutic targets [23, 24]. Furthermore, PCLS can be derived from various species like mice, rats, guinea pigs, sheep, and most importantly humans (hPCLS) [25]. hPCLS can be either generated from diseased or healthy lung tissue. In these cases, disease mechanisms and therapeutic options can be studied. Depending on the size of the lung, several hundred PCLS can be obtained from one lung. Therefore, the use of PCLS also supports the Replacement, Refinement, and Reduction (3R) of animals in relevant studies [26, 27]. Efforts to prolong the viability of PCLS and to optimize their usage have led to improved culture protocols [28–30]. Despite these improvements, there are still challenges with tissue waste and optimal utilization of PCLS because of time constraints and tissue availability [31]. Given the scarcity of human lung tissue samples and the even greater value of samples from diseased patients, an optimized storage protocol for processing large quantities of human PCLS is of high importance for lung research. For these reasons, several groups have developed alternative storage methods, including cryo- [32–34] and cold storage preservation [31]. For both methods, medium changes are not necessary, simplifying handling and shipping of PCLS. This facilitates transport of PCLS between collaborating laboratories, paving the way for setting up PCLS biobanks. Cryopreservation (at -60 to -80 °C), although promising [35], presents risks such as cell damage from ice crystal formation, loss of cell water, and possible degradation of the cell architecture during the freeze-thaw process [4, 36–38]. These risks might be minimized with cryoprotectants like dimethyl sulfoxide (DMSO), which themselves exert disadvantages like cell membrane toxicity and/or oxidative damage [38, 39]. Cold storage preservation at 4 °C is a more “gentle” method, which we have put forward as a viable option using rat PCLS [31]. With a suitable PCLS storage approach, on-demand experiments using tissue from multiple donors could be conducted simultaneously, tissue sharing with other research groups lacking tissue access would be possible and it would also allow the storage of excess PCLS for future experiments and/or for the repetition of experiments in the case of technical problems in downstream assays. Lowering the storage temperature significantly reduces the metabolic activity of the cells, thereby decreasing the energy needed to maintain cellular functions [31], resulting in higher viability over a longer period of time. Additionally, storing PCLS at 4 °C simplifies transport and storage, as it eliminates the need for liquid nitrogen tanks needed for cryopreservation and significantly facilitates shipping since it eliminates the need for dry ice. Originally, cold storage solutions for lung tissue samples were developed from organ transplant storage solutions. Although beneficial, previous studies have described cold-induced injury characterized by mitochondrial dysfunction and oxidative stress [40–44], well-known inducers of cellular senescence. Senescent cells are characterized by a stable cell cycle arrest, an altered metabolic activity, and the secretion of several pro-inflammatory growth factors and cytokines [45]. Moreover, other studies have reported a cold-induced, iron-dependent mechanism of cellular injury, against which the addition of iron chelators showed protective effects [41, 46, 47]. Previously, we tested two cold storage solutions with rat PCLS in 2021 [31], whereas Horn et al. deployed the same solutions for the cold storage preservation of human HepaRG liver spheroids [48]. Therefore, building upon our previous results [31], this study aims to establish the efficacy of the most promising Solution 1 (without iron chelators or with iron chelators, the latter being commercially available as TiProtec^®^) for preserving hPCLS. Here, we demonstrate that TiProtec allows the cold storage of hPCLS for up to 14 days maintaining their viability, metabolic activity, major cellular composition, and tissue integrity as well as their capacity to elicit a fibrotic response. Moreover, we describe for the first-time transcriptional changes associated to cold storage preservation of hPCLS, thereby offering a reference atlas for future studies. Finally, this study contributes to an optimized use of hPCLS, enabling banking, sharing, and on-demand processing and usage of hPCLS for translational lung research.

## Methods

### Aim, design, and setting of the study

To evaluate the effects of long-term cold storage on hPCLS, the special preservation solution TiProtec^®^ and a variant of it, without iron chelators; TiProtec (-), were compared with a standard DMEM/F-12 solution (Table 1). After agarose filling and slicing, 4 mm punches were generated. Slices were kept in culture medium overnight, as previously described and recommended [2, 30]. Accordingly, the day after preparation of hPCLS was defined as baseline (T0) for all experiments. On this day, hPCLS were then transferred to 500 µL of the respective solutions and the samples were stored at 4 °C without medium change until the collection time point (T7, T14, T21, T28). To account for slice-to-slice variability in cellular composition, 4 mm punches derived from the same hPCLS were randomly distributed among the tested cold-storage solutions or baseline stimulations. Prior to functional and stimulation experiments, hPCLS were warmed up for 3 h in standard cell culture conditions (37 °C with 5% CO_2_ and 95% O_2_) in the same cold storage solution in which they were stored respectively, unless otherwise described.

**Table 1:** Composition of DMEM/F-12 and cold storage preservation TiProtec

	DMEM/F-12	TiProtec
Cl^−^	126	103.1
Na^+^	133	16
K^+^	0.4	93
H_2_PO_4_^−^ (41)		1
SO_4_^2−^	0.4	
Mg^2+^	1	8
Ca^2+^	2.22	0.05
Glycine	0.25	10
Alanine	0.05	5
α-Ketoglutarate		2
Aspartate	0.05	5
*N*-Acetylhistidine	0.15	30
Tryptophan	0.05	2
Sucrose		20
Glucose	17.5	10
Hepes Buffer	15	
Deferoxamine*		0.1
LK 614*		0.02
Other compounds	(cf. to Supplementary Table 1)	
pH	7.0-7.6	7.0

Concentrations are given in mmol/L; for a simplified comparison of both solutions the main compounds are displayed here. More detailed information about DMEM/F-12, including other compounds, can be found in the Supplementary Table 1. *Not present in TiProtec (-)

### Ethic statement

The study was approved by the local ethics committee of the Ludwig-Maximilians University of Munich, Germany (Ethic vote 19–630). Written informed consent was obtained for all study participants.

### Precision-cut lung slices

Peritumor control tissue from non-chronic lung diseases (N-CLD) patients (Table 2) were obtained from the CPC-M bioArchive at the Comprehensive Pneumology Center (CPC Munich, Germany). Patients were 53.8% male and had a mean age of 70 ± 7.98 years old. PCLS were generated using either a vibratome HyraxV50 (Zeiss, Germany) or 7000smz-2 Vibratome (Campden Instruments, England) [22, 49]. Human lung tissue was filled with 3% of low gelling temperature agarose in hPCLS culture medium: DMEM/F-12 (Thermo Scientific, USA) with phenol red supplemented with 0.1% Fetal Bovine Serum (FBS), 1% penicillin/streptomycin (P/S) and 1% amphotericin B. Filled tissue was kept at 4 °C at least for 1 h before slicing it into 500 μm slices. Using a biopsy puncher, 4 mm slices were generated and cultured in 24-well plates. After cold storage and for any stimulation assay, hPCLS were cultured for up to 7 days (unless otherwise described) in hPCLS culture medium as described before [22, 49] and the medium was changed every 48/72 h.

**Table 2:** Patient demographics and clinical data

Sex	Age	Smoking status	Tissue Origin
Male	70	ex-smoker	Peritumor
Male	65	ex-smoker	Peritumor
Female	77	never-smoker	Peritumor
Female	65	ex-smoker	Peritumor
Male	83	never-smoker	Peritumor
Female	77	smoker	Peritumor
Male	68	ex-smoker	Peritumor
Male	80	ex-smoker	Peritumor
Male	66	never-smoker	Peritumor
Female	68	ex-smoker	Peritumor
Female	57	ex-smoker	Peritumor
Female	77	ex-smoker	Peritumor
Male	57	smoker	Peritumor
Female	71	ex-smoker	Peritumor
Female	68	ex-smoker	Peritumor

### Materials and chemicals

Low melting point agarose was purchased from Sigma-Aldrich. The Alamar Blue assay and the Live/Dead Staining were performed in DMEM/F-12 (1:1) with 15 mmol/L HEPES and sodium bicarbonate without L-glutamine and phenol red (Sigma-Aldrich, St. Louis, MO/USA; Product ID: D6434-500ML; Table 1). This medium was supplemented with 1% P/S (10,000 units penicillin and 10 mg streptomycin/mL; Sigma-Aldrich). The cold storage solution (TiProtec^®^; Table 1) was either obtained from Dr. Franz Köhler Chemie (Bensheim, Germany) or prepared in the Institute of Physiological Chemistry, Germany, Essen. The iron chelator-free derivative of this solution (TiProtec (-)) was prepared in the Institute of Physiological Chemistry, Essen. The supplement LK 614 (N-hydroxy-3,4-dimethoxy-N-methylbenzamide) was generously provided by Dr. Franz Koehler Chemie, while deferoxamine mesylate was purchased from Novartis Pharma (Basel, Switzerland). Both chelators were freshly added to obtain the final TiProtec. Both, TiProtec and TiProtec (-) were supplemented with 1% P/S. For fibrosis induction, control and fibrotic cocktails (Table 3) were freshly prepared just before the treatment in DMEM/F-12 supplemented with 1% P/S (Sigma-Aldrich), 1% amphotericin B (Sigma, 250 µg/ml), and 0.1% FBS (PAN Biotech) as previously described [2]. Transforming growth factor-β1 (TGF-β1) was obtained from R&D systems (Product ID: 240-B-002/CF), reconstituted in 0.1% Bovine Serum Albumin (BSA, Sigma) dissolved in 4 mM HCl in Phosphate Buffered Saline (PBS) at a concentration of 1 µg/mL, and aliquots were stored at -20 °C. Tumor necrosis factor-α (TNF-α) was obtained from R&D systems (Product ID: 210-TA-CF), reconstituted in PBS at a concentration of 0.1 mg/ml, and aliquots were stored at -20 °C. Platelet-derived growth factor-AB (PDGF-AB) was obtained from Gibco (Product ID: PHG0134), reconstituted in 10 mM Acetic Acid (Honeywell Research Chemicals) at a concentration of 0.1 mg/ml, and aliquots were stored at -20 °C. Lysophosphatidic acid (LPA) was obtained from Cayman Chemical (Product ID: 62215), reconstituted in PBS at a concentration of 10 mM, and aliquots were stored at -20 °C. BSA was obtained from Sigma (Product ID: A7906-100G).

**Table 3:** Composition of control cocktail (CC) and fibrotic cocktail (FC). Transforming growth factor- β1 (TGF- β1), tumor necrosis factor- α (TNF- α), platelet-derived growth factor-AB (PDGF-AB), Lysophosphatidic acid (LPA), bovine serum albumin (BSA)

Reagent	CC	FC
TGF-β1	0.1% BSA in PBS	5 ng/ml
TNF-α	0.1% BSA in PBS	10 ng/ml
PDGF-AB	10 mM Acetic acid	10 ng/ml
LPA	PBS	5 µM

### Viability

**Fig. 1:**
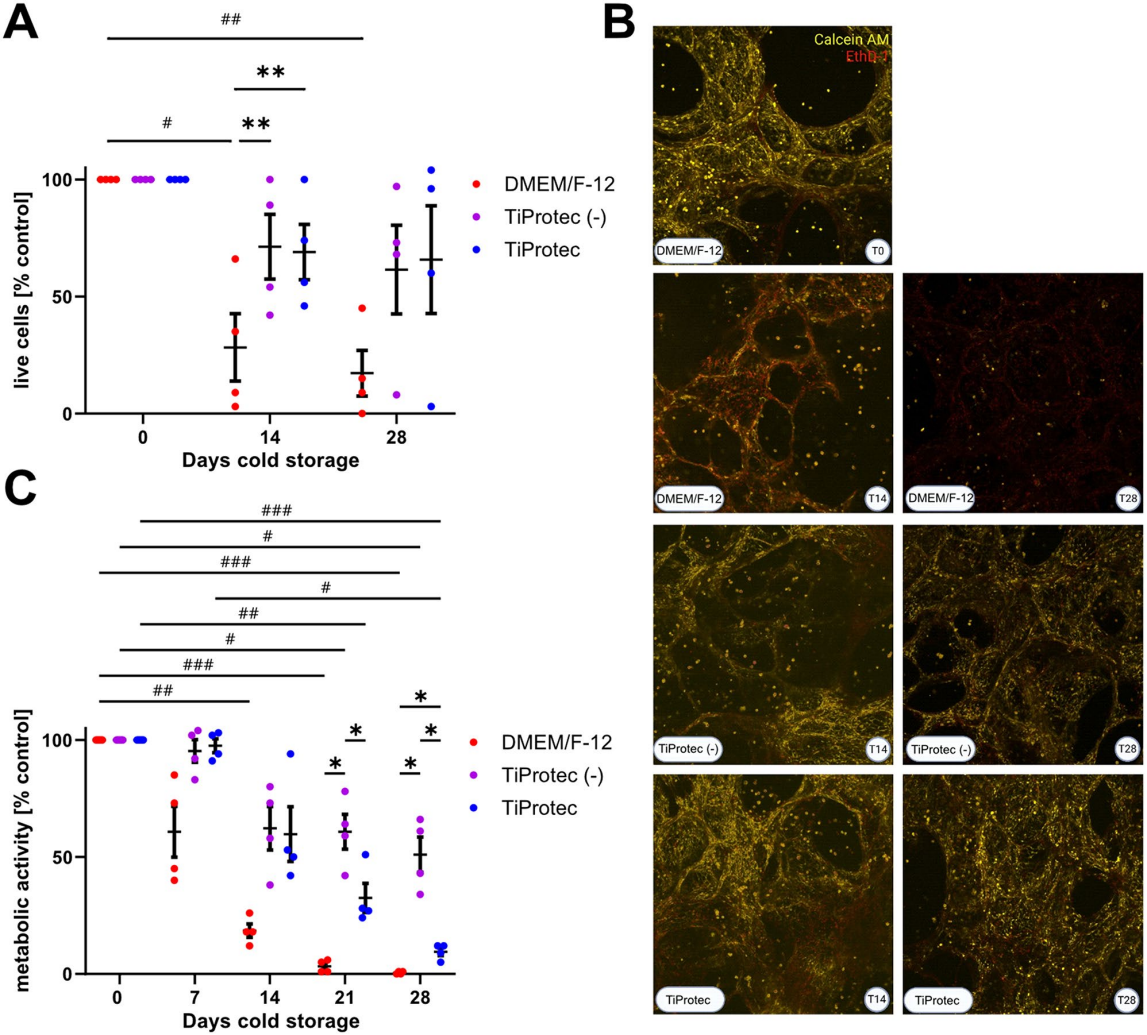
Viability and metabolic activity after long-term cold storage. After preparation, hPCLS were cold stored in DMEM/F-12, TiProtec, or TiProtec (-), at 4 °C for up to 28 days. **A**) Viability changes were assessed by Calcein AM and EthD-1 staining. Viability is expressed as a percentage of the baseline viability of freshly sliced hPCLS at day 0 (100%). A positive control was also measured, after treating hPCLS with 1% Triton-X. Data set was analyzed using a RM two-way ANOVA followed by a Tukey´s multiple comparison test: * simple effects of the medium and # simple effects of storage time. Data are presented as means ± SEM (*n* = 4 patients, single dots represent average activity from 3 technical replicates per patient). Asterisks indicate significant differences (** *p* < 0.01, # *p* < 0.05, ## *p* < 0.01). **B**) Representative images of Live/Dead™ staining of hPCLS at baseline (T0) and after long-term cold storage. **C**) Quantification of Alamar Blue assay. The baseline metabolic activity was measured on day 0 and set as 100%. A negative control with only medium was also included. Metabolic activity changes are presented as a percentage of the baseline activity of freshly cut hPCLS (T0). Data set was analyzed using a Mixed-effects model followed by a Tukey´s multiple comparison test: * simple effects of the medium and # simple effects of storage time. Data are presented as means ± SEM (*n* = 4–6 patients, single dots represent averaged activity from 3 technical replicates per patient). Asterisks indicate significant differences (*p < 0.05, # *p* < 0.05, ## *p* < 0.01, ### *p* < 0.001)

To visualize and quantify possible cold storage induced cell death, the Live/Dead™ Viability/Cytotoxicity Staining kit for mammalian cells containing calcein acetoxymethylester (calcein AM) and ethidium homodimer-1 (EtHD-1) from Invitrogen (Thermo Fisher Scientific, Karlsruhe, Germany) was used in combination with confocal microscopy. The cold storage duration was set between 14 and 28 days, based on preliminary experiments that indicated substantial changes in metabolic activity within this interval (Fig. 1C) and to enable a direct comparison to the results of Tigges et al. [31]. Living cells fluoresced green due to the enzymatic cleavage of calcein AM by intracellular esterases, while dead cells fluoresced red as EthD-1, as a cell impermeable dye, binds to the cell nuclei following the loss of plasma membrane integrity [50]. Measurements were generally performed in triplicates of hPCLS for each solution after a rewarming period of 3 h at T14 and T28 after cold storage in the respective solutions. Baseline measurements (T0) and positive controls, for which 1% Triton-X was applied to one hPCLS per patient, were included. The Live/Dead™ Staining kit was used according to the manufacturer’s instructions, which involved applying 4 µmol/L calcein AM and 4 µmol/L EthD-1 for staining in DMEM/F-12 for 45 min at room temperature on a vibratory plate in the dark. Then, hPCLS were washed three times with Dulbecco’s Phosphate Buffered Saline without Calcium and Magnesium (DPBS; Thermo Fisher Scientific). Before microscopy, hPCLS were weighted with steel wires to prevent floating during examination with a confocal microscope (Leica DMi-8; Leica Microsystems, Wetzlar, Germany) using a 10x objective. The excitation/emission maxima were 503/565 nm for calcein AM and 592/752 nm for EthD-1. Z-stacks and 3D images were generated, consistently applying the same settings. ImageJ was then used to quantify the live and dead cells. Afterwards, percentages of live/dead cells were calculated at the different collection time points using hPCLS treated with Triton-X as maximum cell death and T0 as 100% viability for each donor.

### Metabolic activity

The metabolic activity of hPCLS was determined using an Alamar Blue assay (Invitrogen, Carlsbad, CA/USA). This assay measures the fluorescence intensity of resorufin, which is produced by the reduction of resazurin in live cells as described previously by Tigges et al. [31]. In brief, one hPCLS per solution was placed into a 24-well plate containing 445 µL/well of prewarmed DMEM/F-12. Then, 5 µL of Alamar Blue reagent were added to each well. Afterwards the hPCLS were incubated for 2 h at 37 °C on a vibratory plate. Subsequently, 125 µL of the supernatant were transferred in technical duplicates into a 96-well plate. Control wells, which only contained DMEM/F-12 and Alamar Blue, were treated in the same way as described above and measured in technical duplicates. Fluorescence intensity was measured with a multimode microplate reader (Spark, Tecan Group Ltd., Männedorf, Switzerland), with excitation and emission wavelengths set as 560 nm and 590 nm, respectively. Background fluorescence, represented by the control values, was subtracted from the readings. The measurements were taken at T7, T14, T21, and T28 in hPCLS triplicates for each solution after a rewarming period of 3 h. These timepoints were chosen, since a direct comparison of the results to the ones of Tigges et al. [31] was planned to study whether there were species induced differences. The fluorescence intensity of the various long-term cold-stored hPCLS was then compared with the baseline fluorescence activity measured at T0.

### Formalin-fixed paraffin-embedding of PCLS

hPCLS were washed once with 1X PBS, fixed in 4% PFA solution for 1 h at 37 °C, and kept at 4 °C in 1X PBS until used. Fixed hPCLS were transferred to embedding cassettes and processed by a Microm STP 420D Tissue Processor (Thermo Scientific, USA) with the following steps: 50% EtOH (1 cycle, 60 min), 70% EtOH (1 cycle, 60 min), 96% EtOH (2 cycles, 60 min each), 100% EtOH (2 cycles, 60 min each), Paraffin (1 cycle, 30 min), and Paraffin (3 cycles, 45 min each). Then, tissue was embedded in paraffin Type 3 (Thermo Scientific, USA) using the Modular Tissue Embedding Center EC 350 (Thermo Scientific, USA), and blocks were kept at 4 °C until use. Formalin-fixed, paraffin-embedded (FFPE) sections were cut using a Hyrax M55 microtome (Zeiss, Germany) mounted on slides, dried overnight at 40 °C, and kept at 4 °C until use.

### Hematoxylin and eosin staining

Hematoxylin and Eosin (H&E) staining was performed to assess morphological changes in hPCLS after cold storage in DMEM/F-12, TiProtec, or TiProtec (-). Samples were collected at T7, T14, T21, and T28 and FFPE sections were generated. FFPE sections were dried at 60 °C for 30 min to 1 h. Then, they were deparaffinized with the following steps: Xylene (2 cycles, 5 min each), 100% EtOH (2 cycles, 3 min each), 90% EtOH (1 cycle, 3 min), 80% EtOH (1 cycle, 3 min), 70% EtOH (1 cycle, 3 min), and Milli-Q water (1 cycle, 5 min). Then, FFPE sections were stained with hematoxylin for 10 min, rinsed, and then stained with eosin for 45 s followed by final washing step with Milli-Q water. Samples were dehydrated prior to the mounting and then they were imaged with an AxioImager (Zeiss) at 20X magnification.

### Immunofluorescence

FFPE sections were deparaffinized and exposed to heat-induced antigen retrieval (10 mM citrate buffer, pH = 6.0, 1 cycle at 125 °C for 30 s, 1 cycle at 90 °C for 10 s). Slides were washed twice with 1X PBS and blocked for 1 h at RT in a humid chamber with 10% Normal Donkey Serum in DAKO Antibody Diluent (Agilent Technologies, USA). Samples were incubated with primary antibodies (Table 4) diluted in DAKO Antibody Diluent (Agilent Technologies, USA) and incubated at 4 °C overnight in a humid chamber. The next day, samples were washed twice in 1X PBS for 5 min and incubated with secondary antibodies (Table 4, diluted 1:250) and DAPI (1:500) prepared in 1% BSA in 1X PBS and incubated for 2 h at RT in a humid chamber. hPCLS were blocked with the Vector^®^ TrueVIEW^®^ Autofluorescence Quenching Kit (Vector laboratories, USA) for 2–3 min at RT in a humid chamber. Finally, hPCLS were mounted using DAKO Fluorescence mounting medium (Agilent Technologies, USA) and imaged using an AxioImager (Zeiss) acquiring at least 3 regions of interest per sample at 20X. Fiji (version 1.53t) was used for automatic quantification of mean fluorescence intensity or cell percentage, which were normalized to cell count based on DAPI signal.

**Table 4:** Antibodies used for immunofluorescence staining

Target Protein	Host	Company	Ref. No
P21	rabbit	Abcam	ab109520
PDPN	sheep	R&D system	AF3670
aSMA	mouse	Millipore Sigma	A5228
FN1	rabbit	Santa cruz	sc-9068

### Determination of baseline senescence

To determine whether the three solutions induce cold storage-related cellular senescence in human PCLS, samples were collected at T0, T7, T14, and T21. These time points were chosen to compare baseline senescence with changes in metabolic activity. For this, 4 mm punches were prepared and stored in DMEM/F-12, TiProtec (-), or TiProtec for the listed time points. At collection points, samples were washed once with 1X PBS and prepared into FFPE sections as previously described. Immunofluorescence was used to determine the expression of the Cyclin Dependent Kinase Inhibitor 1 A (CDKN1A/P21) as a marker for cellular senescence and Podoplanin (PDPN) as a lung structural marker of the alveolar region (Table 4).

### Induction of fibrosis

As previously described [2], hPCLS were treated with a control (CC) or fibrotic cocktail (FC) (Table 3) at baseline (T0) or after cold storage in TiProtec solutions (T7, T14). These time points were selected as our findings suggested tissue viability and function were most effectively maintained for up to 14 days of cold storage. For this, both cocktails were prepared in DMEM/F-12 with phenol red supplemented with 0.1% FBS, 1% P/S and 1% amphotericin B. After cold storage and a 3 h rewarming period, hPCLS were washed once with pre-warmed DPBS and then the CC/FC cocktails were added. hPCLS were kept in standard cell culture conditions (37 °C, 5% CO_2_) and treatment was replenished after two days. Samples were collected at day 5 after treatment. On the collection day, six 4 mm punches per condition were flash frozen in liquid nitrogen and the corresponding supernatants were collected and kept at -80 °C until use. Moreover, two 4 mm punches were collected for immunostaining, as previously described.

### RNA isolation

For RNA isolation we adapted the protocol previously published [51]. In summary, six 4 mm hPCLS were washed once with cold 1X DPBS and snap frozen in liquid nitrogen either at baseline, after cold storage, or after stimulation. Then, 400 µl of TRIzol™ and one 5 mm stainless steel bead (Qiagen, USA) were added to each sample and tissue was homogenized using a TissueLyser II (Qiagen, USA) 3 times at 27 Hz for 1 min. RNA was then precipitated using 100% EtOH, loaded into RNeasy MinElutespin columns, and spun down at full speed (16,000 x g/RFC) for 30 s at RT. Samples were washed once with RW1 buffer and treated with DNAseI diluted in RDD buffer, according to the manufacturer´s instructions (Qiagen, USA). Samples were then sequentially washed with RW1 buffer (12,000 x *g*/RFC for 30 s, RT), RPE Buffer (12,000 x *g*/RFC for 30 s, RT), and 80% EtOH (12,000 x *g*/RFC for 2 min, RT). Columns were air dried at full speed (16,000 x *g*/RFC) for 5 min at room temperature (RT) and RNA was eluted in 20 µl RNase-free water. RNA concentration was determined using a Nanodrop with an average yield of 34.06 ± 10.54% ng/µl (T0), 28.96 ± 10.60% ng/µl (T7 DMEM/F-12), 34.43 ± 24.94% ng/µl (T7 TiProtec (-)), 31.33 ± 6.375 ng/µl (T7 TiProtec), 35.39 ± 12.70% ng/µl (T14 TiProtec (-)), and 44.78 ± 28.68 ng/µl (T14 TiProtec), with not statistical significant differences among conditions as tested by Kruskal-Wallis test, followed by a Dunn´s multiple comparisons test. RNA was stored at -80 °C until further use.

### RT-qPCR

500–1000 ng RNA, as determined by Nanodrop quantification, were denatured in 20 µl of RNAse-free water (15 min at 70 °C). Then, the cDNA reaction mix (Table 5) was added to each sample and incubated for one cycle at 20 °C for 10 min, one cycle at 43 °C for 75 min, and one cycle at 99 °C for 5 min. Finally, cDNA was diluted with RNAse-free water and kept at -20 °C until use. The RT-qPCR reaction mix was prepared using Luna^®^ Universal qPCR Master Mix and the desired primer pair (final concentration 5 µM) (Table 6). Samples were loaded in a 96-well plate and incubated in a QuantStudio3 (Thermo Fisher Scientific) following these steps: Pre-incubation: (1 cycle) 50 °C for 2 min. Denaturation: (1 cycle) 95 °C for 10 min. Amplification: (40 cycles) at 95 °C for 3 s and 60 °C for 30 s. Melting curve: (1 cycle) 95 °C for 15 s, 60 °C for 1 min and 95 °C for 15 s. A two-derivative analysis was used to determine Ct values and the 2–∆∆Ct method [52] was used to calculate the relative fold gene expression.

**Table 5:** Mastermix for cDNA synthesis

Reagent	Article ID	Final concentration
Random Hexamers	N8080127	10 µM
dNTP Mix	R0192	2 mM
5X First– Strand Buffer	18057018	1X
0,1 M DTT	18057018	40 mM
Reverse Transcriptase	28025013	10 U/µl
RNAse Inhibitor	N8080119	4 U/µl

**Table 6:** Primers for RT-qPCR

Primer	Sequence (5’-3’)
*FN1*_fw	CCGACCAGAAGTTTGGGTTCT
*FN1*_rv	CAATGCGGTACATGACCCCT
*ACTA2*_fw	CGAGATCTCACTGACTACCTCATGA
*ACTA2*_rv	AGAGCTACATAACACAGTTTCTCCTTGA
*COL1A1*_fw	CAAGAGGAAGGCCAAGTCGAG
*COL1A1*_rv	TTGTCGCAGACGCAGATCC
*HPRT*_fw	AAGGACCCCACGAAGTGTTG
*HPRT*_rv	GGCTTTGTATTTTGCTTTTCCA

### RNA-bulk-sequencing

Bulk RNA was isolated as described previously from three (DMEM/F-12) and four (TiProtec solutions) different biological replicates at T0 and after 7 and 14 days in cold storage. Time points were selected accordingly to viability and metabolic activity of cold-stored PCLS. Samples were sent to Novogene for quality control, library preparation, and sequencing. RNA quantity and quality was determined using a Bioanalyzer, with no significant differences found among conditions. Messenger RNA was purified from total RNA using poly-T oligo-attached magnetic beads. After fragmentation, the first strand cDNA was synthesized using random hexamer primers. Then, the second strand cDNA was synthesized using dUTP, instead of dTTP. The directional library was ready after end repair, A-tailing, adapter ligation, size selection, USER enzyme digestion, amplification, and purification. The library was checked with Qubit and real-time PCR for quantification and bioanalyzer for size distribution detection. Quantified libraries were pooled and sequenced on Illumina NovaSeq X Plus Series (PE150) platforms, according to effective library concentration and data amount. The gene annotation used for quantification was Ensembl version 108. Quality controls were performed in R (4.2.3) and RStudio (2023.03.0). Raw counts were corrected for batch bias due to biological variance using ComBatseq function in the sva package (3.46.0). Then, differential expression analysis was done using the DESeq2 package (pAdjustMethod = ”BH”, alpha = 0.05) and shrinkage of the Log-fold change (LFC) [53]. Differentially expressed genes (adjusted *p*-value < 0.05, LFC > 0) were extracted and used for initial exploration and cell type signature enrichment analysis. Differentially expressed genes (adjusted *p*-value < 0.05, LFC > 1) were extracted and used for gene set enrichment analyses using the fsgea (1.24.0), DOSE (3.24.2), and Cluster Profiler (4.6.2) packages. Cell type signature enrichment analysis was performed as described in [54, 55], using the top 100 marker genes for each cell type of the publicly available single-cell RNA-seq hPCLS dataset [23]. The enrichment scores (-log10 p-values signed by effect size) reflect either enrichment or depletion of the respective cell types, with positive values indicating enrichment and negative values indicating cell type depletion.

### Statistics

All datasets were tested for normality using a Shapiro-Wilk test. Data derived from viability, metabolic activity, and baseline senescence was analyzed using a two-way ANOVA model or Mixed-effect model followed by Tukey`s multiple comparisons test. Data from RT-qPCR after fibrotic stimulation was analyzed using an unpaired t-test (baseline) or a Kruskal-Wallis test followed by a Dunn´s multiple comparisons test (day 7 and day 14). Data from immunofluorescence quantification after fibrotic stimulation was normalized to control cocktail samples and analyzed with one sample t-test. Differences with p-values < 0.05 were considered significant. Data are reported as the mean and standard deviation or standard error of the mean. Single points represent independent biological replicates. All data and graphs were analyzed and generated in GraphPad Prism v.10.0.2.

## Results

### TiProtec and TiProtec (-) preserve hPCLS viability for more than 14 days

Live/Dead™ staining was performed to assess the cell viability of hPCLS at baseline and after cold storage in DMEM/F-12, TiProtec, and TiProtec without the iron chelators LK 614 and deferoxamine (TiProtec (-)). The baseline cell viability (T0) was measured one day after slicing prior to long-term cold storage. Residual viability after cold storage, expressed as a percentage of the initial viability (100%) was determined after 7, 14, and 28 days of cold storage. In general, hPCLS stored in TiProtec and TiProtec (-) exhibited higher viability than hPCLS stored in DMEM/F-12 (Fig. 1A, B). Preservation of cell viability was time- (*p* = 0.0281) and medium- (*p* = 0.0050), but not patient-dependent. When stored in DMEM/F-12 the viability significantly dropped to 28 ± 25% (*p* = 0.0312) by day 14, and further decreased to 17 ± 17% (*p* = 0.0070) by day 28 (Fig. 1A). In contrast, cold storage in both TiProtec variants did not result in significant viability declines throughout the entire cold storage period (T14: TiProtec (-) = 71 ± 24%, TiProtec = 69 ± 21%; T28: TiProtec (-) = 62 ± 33%, TiProtec = 65 ± 40%). The presence of iron chelators did not significantly affect cold-stored hPCLS viability. TiProtec (-) showed a slight benefit on day 14, while TiProtec had a benefit on day 28. Overall, hPCLS stored in TiProtec with and without iron chelators remained more viable than those in DMEM/F-12 after 14 days (Fig. 1A, B). A similar pattern was seen at 28 days, but the effect was less pronounced. In conclusion, cold storage in TiProtec and TiProtec (-) maintains cellular viability for up to 28 days, with no significant impact from the addition of iron chelators. Accordingly, H&E staining also indicated that tissue integrity is preserved up to 28 days with both cold storage solutions (Suppl. Figure 1).

### TiProtec and TiProtec (-) maintain the metabolic activity for up to 14 days

Metabolic activity was measured at baseline (T0 = 100%) and after cold storage (T7, T14, T21, T28). The metabolic activity was dependent on the duration of storage (*p* < 0.0001) and type of storage solution (*p* = 0.0004). hPCLS stored in TiProtec and TiProtec (-) had a higher metabolic activity than those stored in DMEM/F-12 (Fig. 1C). Indeed, the metabolic activity of hPCLS had a decline already after 7 days of cold storage in DMEM F-12 that continuously decreased over the storing time (Fig. 1C), becoming significant after 14 days with 19 ± 5% (*p* = 0.01), and further decreasing to 3 ± 2% (*p* < 0.001) after 21 days, and to 1 ± 1% (*p* < 0.01) after 28 days. These results indicate that DMEM/F-12 is unsuitable for long-term (> 7 days) cold storage of hPCLS. In contrast, TiProtec and TiProtec (-) performed better, as metabolic activity was not significantly reduced until day 21. Up to 14 days of cold storage, the presence of iron chelators did not have a significant effect on the metabolic activity, as this was comparable for both solutions: TiProtec (-) 62 ± 16%; TiProtec 60 ± 20% (Fig. 1C). The addition of iron chelators (TiProtec) appears not to be beneficial to improve the metabolic activity after cold storage of hPCLS longer than 14 days, as the metabolic activity was significantly lower in its presence: TiProtec (-) 61 ± 13% (*p* = 0.0395), TiProtec 33 ± 11% (*p* = 0.0051) after 21 days and TiProtec (-) 51 ± 13% (*p* = 0.0221), TiProtec 10 ± 3% (*p* < 0.001) after 28 days. TiProtec (-) demonstrated superior performance in maintaining the metabolic activity of hPCLS between days 21 and 28 compared to TiProtec. In conclusion, both versions of TiProtec effectively preserved metabolic activity during cold storage, with minimal loss observed up to 7 days and no significant decline up to 14 days, whereas it was significantly altered from day 21 onwards.

### hPCLS stored in TiProtec and TiProtec (-) preserve transcriptional and cellular identity of fresh hPCLS

**Fig. 2:**
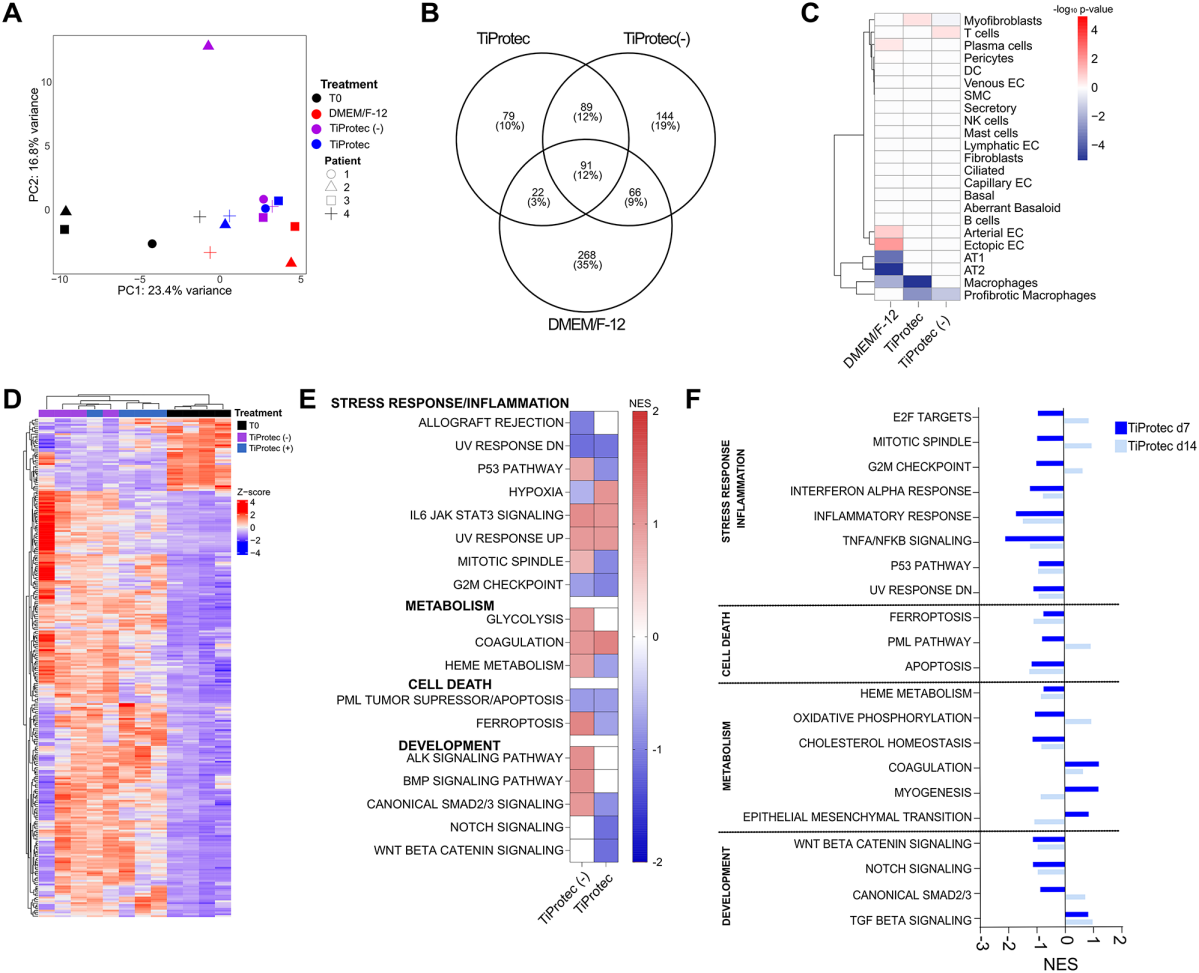
Transcriptional changes in hPCLS after cold storage. **A**) Principal component analysis for freshly cut hPCLS (T0) and after 7 days of cold storage in DMEM/F-12, TiProtec (-), and TiProtec. Shapes show three (DMEM/F-12) or four (T0, TiProtec (-), TiProtec)) different biological replicates and colors indicate the cold storage solution. **B**) Venn diagram of differentially expressed genes (DEG) after 7 days of cold storage when compared to T0 baseline control (LFC > 0). **C**) Cell type signature enrichment analysis of main cellular compartments in hPCLS based on DEG displayed in B. **D**) Heatmap of DEG after 7 days of cold storage in TiProtec (-) or TiProtec when compared to T0 baseline control (LFC > 1). **E**) Deregulated pathways in hPCLS after 7 days of cold storage in TiProtec or TiProtec (-) based on DEG from D. **F**) Deregulated pathways in hPCLS after 7 and 14 days of cold storage in TiProtec based on DEG in comparison to T0 control (LFC > 1).

We have shown for the first time that hPCLS can be stored at least for 14 days at 4 °C without significant changes in viability or metabolic activity. Therefore, we next used bulk-RNA sequencing to characterize the molecular and cellular changes occurring in hPCLS after cold storage in DMEM/F-12 or TiProtec with and without iron chelators for 7 and 14 days when compared to freshly cut hPCLS (T0). First, we performed a principal component analysis (PCA) to visualize and identify the most important factors driving the differences among the evaluated conditions [56]. Here, we found that at day 7, the principal component 1 (PC1) explained 23.4% of the variance indicating differences between freshly cut hPCLS (black symbols) and cold-stored hPCLS (Fig. 2A). Moreover, PC2 explained additional 16.8% of the variance indicating differences among the three cold-storage solutions used (Fig. 2A). Similar sample separation was observed after 14 days, with PC1 explaining 40.8% of the total variance indicating differences depending on cold storage solution (Suppl. Figure 2A). After differential expression analysis, we found that hPCLS stored in DMEM/F-12 for 7 days had the most dramatic transcriptional changes with 447 (3.1% of total genes) differentially expressed genes (DEG) vs. 390 (2.7% of total genes) DEG for TiProtec (-) and 281 (2.0% of total genes) DEG for TiProtec when compared to freshly sliced hPCLS (Fig. 2B). After 14 days, hPCLS cold-stored in TiProtec also displayed the lowest amount of DEG with 297 (2.1% of total genes) vs. 453 (3.1% of total genes) for TiProtec (-) (Suppl. Figure 2B). We next performed a cell type signature enrichment analysis using the top 100 genes for each cellular compartment derived from a single-cell reference atlas previously described for hPCLS [23]. For this, the cell signatures from all cells in hPCLS are used to predict how much each cell type contributes to the global gene expression changes observed in our dataset [54, 55]. Here we observed that cold storage in DMEM/F-12 led to significant transcriptional changes in several cellular compartments of hPCLS, including a relative loss of epithelial cells (Fig. 2C). Storage in TiProtec (-) or TiProtec affected fewer cellular compartments including a predicted relative loss of pro-fibrotic immune cells and myofibroblasts after 7 days (Fig. 2C) and enrichment of mesenchymal cells after 14 days (Suppl. Figure 2C). To further characterize molecular changes in cold-stored hPCLS, we performed an unsupervised hierarchical clustering analysis based on the DEG for TiProtec (-) and TiProtec in comparison to T0 baseline control [56]. Here, we observed that samples stored in TiProtec have a transcriptional profile that is closer to freshly cut hPCLS than those stored in TiProtec (-) (Fig. 2D) with a similar trend after 14 days of cold storage (Suppl. Figure 2B). This together suggests that cold storage in TiProtec or TiProtec (-) maintains not only the viability and metabolic activity of hPCLS but also the transcriptional signature of fresh hPCLS for 14 days. Finally, a gene set enrichment analysis (GSEA) for different molecular pathways showed that cold storage in TiProtec (-) upregulated processes related to stress response, cell death, and development, while TiProtec downregulated most of these pathways (Fig. 2E). Comparison of different pathways after 7- and 14-days cold storage in both TiProtec solutions revealed a sustained downregulation of pathways associated to stress response, inflammation, and cell death in TiProtec (Fig. 2F, Suppl. Figure 3B). The main differences over time in hPCLS stored in TiProtec were an increase of cell cycle and the decrease in mesenchymal activation pathways at day 14 (Fig. 2F), while hPCLS stored in TiProtec (-) showed activation of these pathways already at day 7 (Suppl. Figure 4). In conclusion, TiProtec allows the cold storage of hPCLS for up to 14 days preserving gene expression signatures of cold-stored hPCLS and down-regulating pathways associated with stress response, cell death, and inflammation providing a long-term protective effect in hPCLS from cold storage-induced inflammation and oxidative stress.

### TiProtec and TiProtec (-) prevent inflammation, hypoxia, and maintain cellular composition in hPCLS

**Fig. 3:**
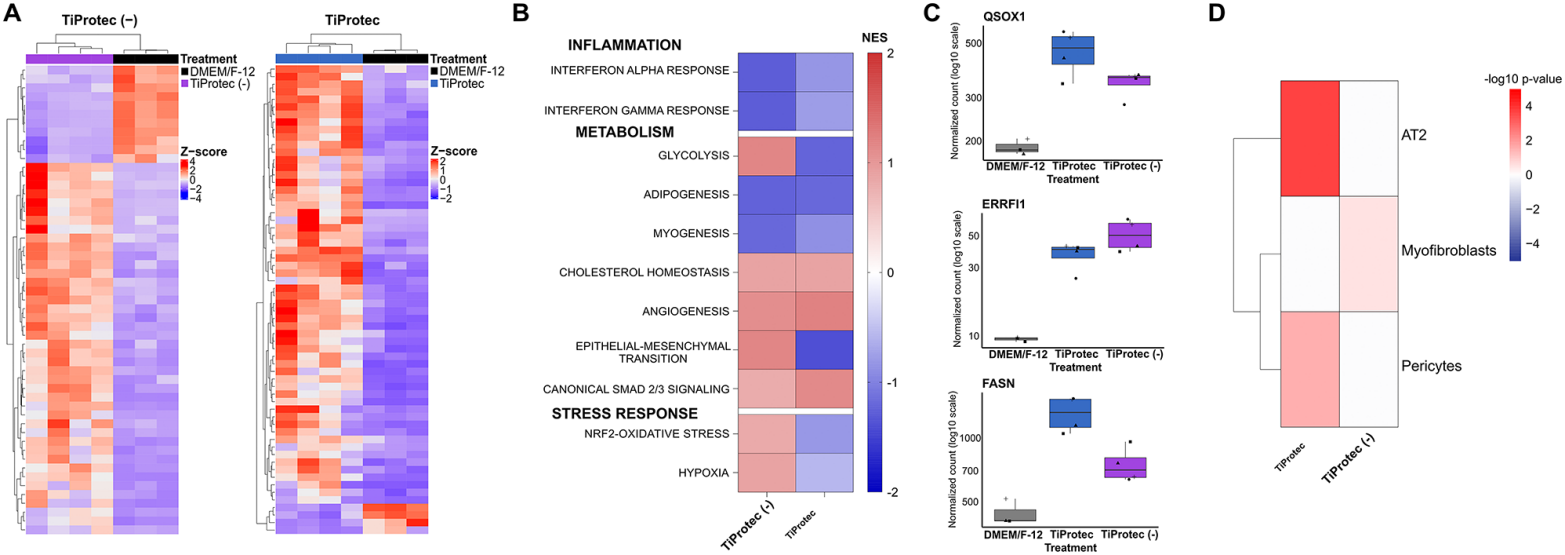
Transcriptional changes induced by TiProtec and TiProtec (-) in comparison to DMEM/F-12 after 7 days of cold storage. **A**) Heatmap of differentially expressed genes (DEG) after 7 days of cold storage when compared to DMEM/F-12 in hPLCS obtained from three (DMEM/F-12) or four (TiProtec (-), TiProtec) different donors. **B**) Gene set enrichment analysis of deregulated pathways in hPCLS after cold storage based on DEG from A. **C**) Normalized counts of up- and -downstream regulators of oxidative stress-associated pathways after 7 days of cold storage in DMEM/F-12, TiProtec (-), or TiProtec. Single points with different shapes represent three (DMEM/F-12) or four (TiProtec solutions) different biological replicates. **D**) Cell type signature enrichmentanalysis in hPCLS after cold storage with TiProtec or TiProtec (-) when compared to DMEM/F-12 for 7 days

Then, we investigated the transcriptional changes associated with a better cold storage preservation of TiProtec and TiProtec (-) in comparison to DMEM/F-12. For this, we extracted the DEG of hPCLS after 7 days of cold storage in both solutions using DMEM/F-12 as reference. Here, we found that TiProtec shows a different profile than TiProtec (-) (Fig. 3A). Gene set enrichment analysis showed that both solutions downregulate interferon-associated inflammatory response in hPCLS (Fig. 3B). However, TiProtec seems much more effective in downregulating other metabolic functions as well as oxidative-stress response and hypoxia in comparison to TiProtec (-) (Fig. 3B), as shown by a stronger regulation of up- and downstream regulators of these pathways including Quiescin sulfhydryl oxidase 1 (*QSOX1*) and Fatty Acid Synthase (*FASN*) after cold-storage in TiProtec (Fig. 3C). Finally, cold storage in TiProtec preserved the identity of cellular populations such as alveolar type II cells and pericytes, both being one of the most susceptible and commonly altered cell types in cultured hPCLS (Fig. 3D) [23]. This together supports the idea that TiProtec allows long-term cold storage of hPCLS by preventing inflammation and oxidative stress responses and preserving vulnerable cell types (up to 14 days).

### Cold storage does not induce cellular senescence in hPCLS

**Fig. 4:**
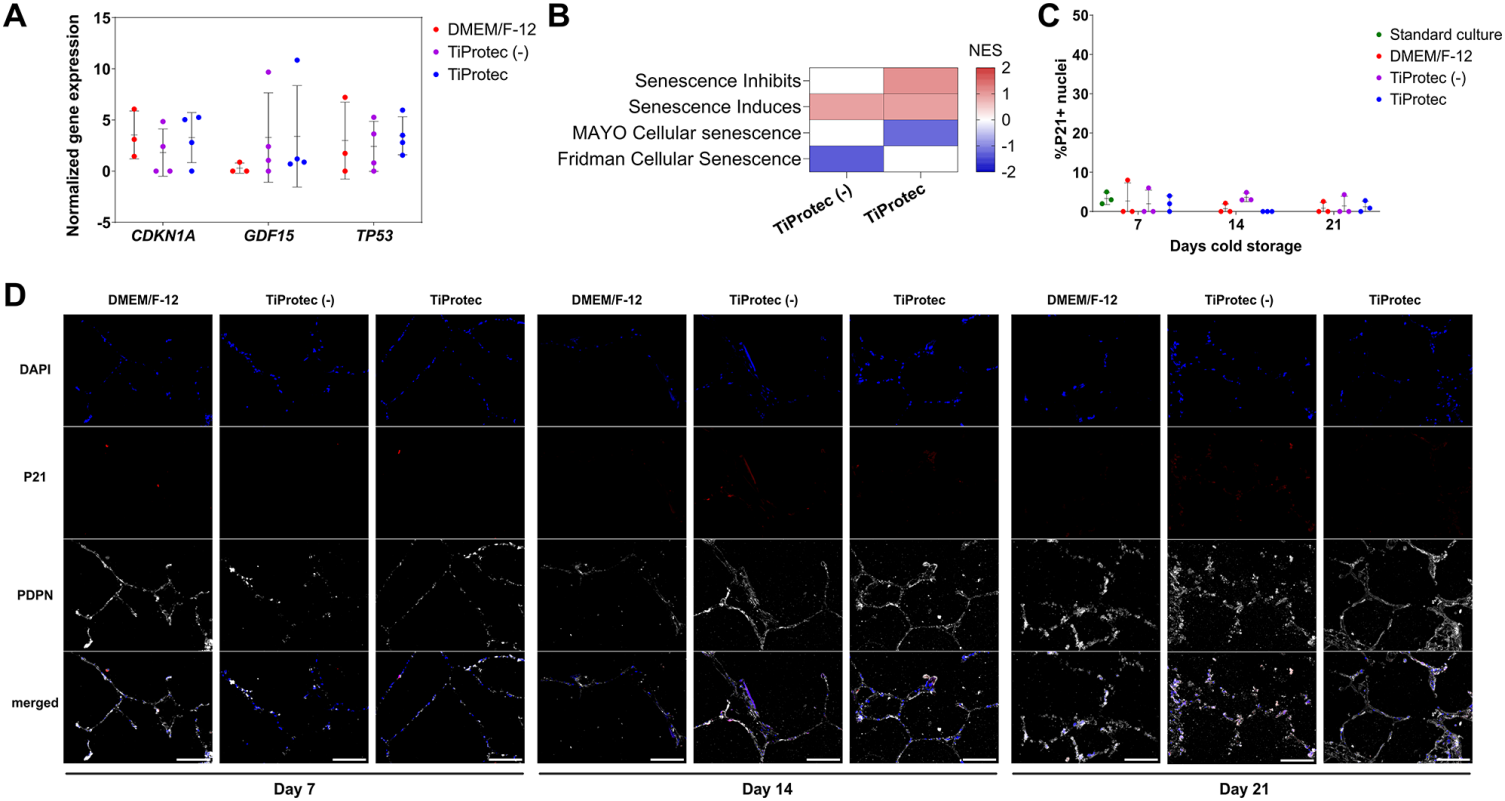
Senescence response in cold-stored hPCLS. **A**) Normalized counts for senescence-associated genes: *CDKN1A/P21*, *GDF-15*, and *TP53*. Single points represent independent donors (N = three (DMEM/F-12)) or four (T0, TiProtec (-), TiProtec)). **B**) Heatmap of enrichment scores after GSEA for senescence-related pathways of DEG from day 7 cold storage in TiProtec (-) or TiProtec in comparison to DMEM/F-12. **C**) Quantification of P21/CDKN1A + nuclei in hPCLS after standard cell culture for 7 days or cold storage in DMEM/F-12, TiProtec (-), or TiProtec for 7, 14, and 21 days. Single points represent independent donors (*N* = 3). **D**) Representative images of immunostaining for P21/CDKN1A and Podoplanin (PDPN, structural marker for alveolar region) in hPCLS after cold storage in DMEM/F-12, TiProtec (-), or TiProtec for 7, 14, and 21 days. Scale bar = 100 μm.

Given our previous results, suggesting a protective effect of cold storage for stress-induced inflammatory response, in both TiProtec solutions, we aimed to determine whether TiProtec or TiProtec (-) protected hPCLS from cold storage induced-senescence. For this, we first explored the gene expression of senescence-associated markers, cyclin dependent kinase inhibitor 1 A (*CDKN1A/P21*), tumor suppressor 53 (*TP53*), and growth differentiation factor 15 (*GDF-15*), in our dataset after 7 days of cold storage in both TiProtec variants in comparison to DMEM/F-12. Here we did not find significant differences in the expression of the evaluated genes (Fig. 4A). Moreover, a gene set enrichment analysis revealed an increase of pathways linked to senescence induction but no change in widely accepted senescence gene lists including SenMayo [57] (Fig. 4B). These results were also confirmed in situ, where we evaluated the nuclear protein levels of CDKN1A/P21 after 7, 14, and 21 days of cold storage in DMEM/F-12, TiProtec, or TiProtec (-) and compared it to standard culture at 37 °C, CO_2_ for 7 days. Here, we observed similarly low levels of CDKN1A/P21 expression after cold storage in comparison to standard cell culture of hPCLS (Fig. 4C, D), suggesting that cold storage does not induce cellular senescence in hPCLS, and this effect does not depend on the presence of chelators.

### Cold-stored hPCLS can be used in a clinically relevant fibrosis model

**Fig. 5:**
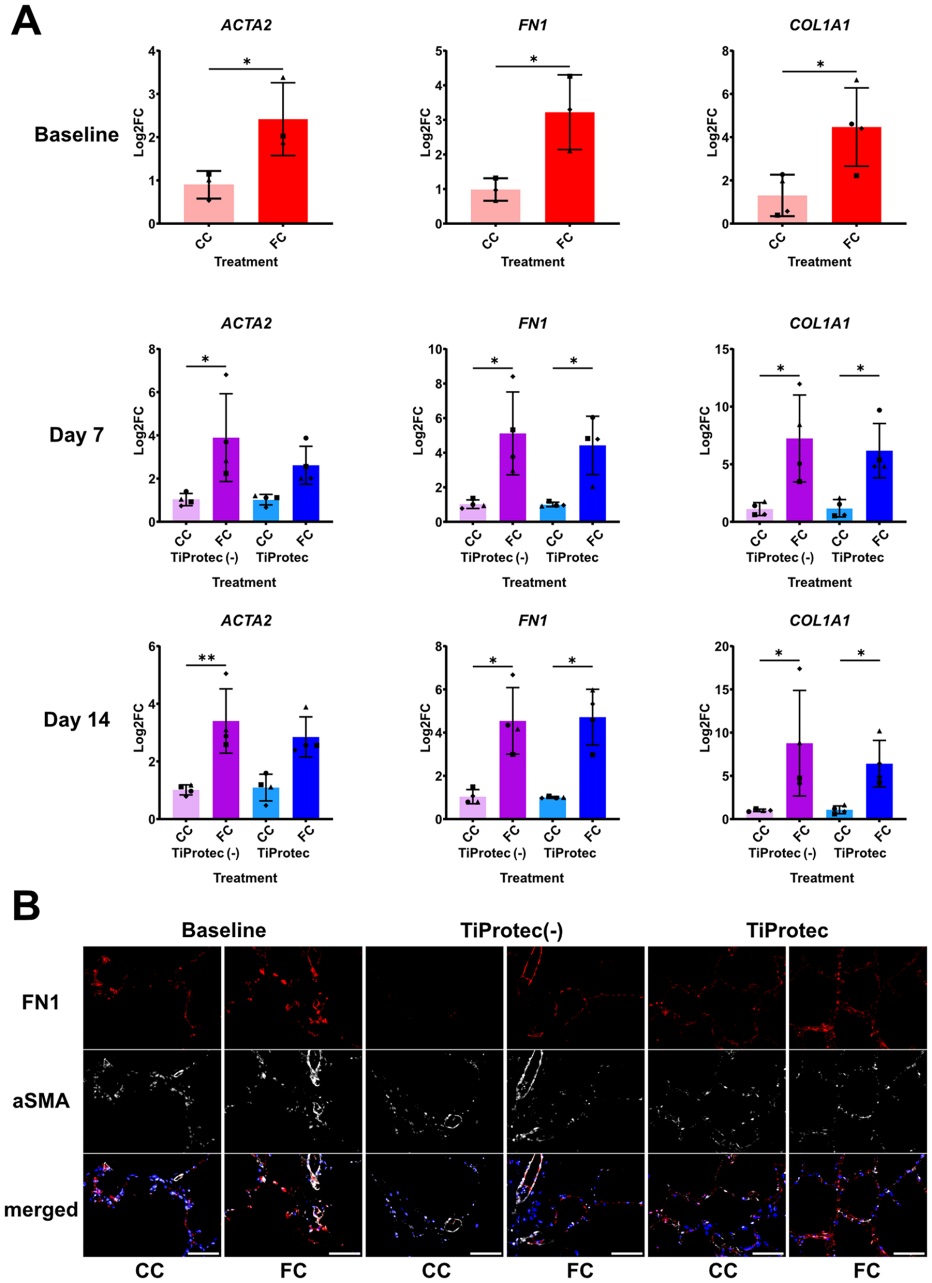
Evaluation of early-fibrotic changes in cold-stored hPCLS after fibrotic stimulation. **A**) RT-qPCR of the extracellular matrix-related genes *COL1A1* and *FN1* and the myofibroblasts marker, *ACTA2*, in hPCLS treated with control (CC) or fibrotic cocktail (FC) at baseline or after 7 and 14 days of cold storage with TiProtec or TiProtec (-). Single points with different shapes represent independent biological replicates (*N* = 4). * *p* < 0.05, ** *p* < 0.01 after unpaired-t-test (baseline) or Kruskal-Wallis test followed by Dunn´s multiple comparison test (day 7, day 14). **B**) Representative images of immunostaining for fibrosis-related proteins (aSMA and FN1) in hPCLS treated with CC or FC at baseline or after 7 days of cold storage with TiProtec or TiProtec (-). Scale bar = 100 μm.

Human PCLS have been widely used to model and study chronic lung diseases such as idiopathic pulmonary fibrosis (IPF) [2, 49, 58]. Among them, the use of a fibrotic cocktail induces structural and cellular changes in hPCLS recapitulating early stages of lung fibrosis characterized by upregulation of alpha smooth muscle actin (*ACTA2*, aSMA) a marker for activated myofibroblasts, as well as extracellular matrix proteins like Fibronectin (*FN1*) and Collagen 1A1 (*COL1A1*) after 5 days of treatment [2]. Therefore, we were interested in evaluating whether cold-stored hPCLS retain their capacity to response to pro-fibrotic stimuli. To this end, we exposed hPCLS to control (CC) or fibrotic (FC) cocktail directly after slicing (T0 baseline) or after being cold stored in TiProtec (-) or TiProtec for 7 and 14 days. hPCLS stored in TiProtec (-) and TiProtec retained their fibrotic response even after 14 days of cold storage as shown by higher gene expression of *ACTA2*, *FN1*, and *COL1A1* after FC treatment in comparison to CC as observed at baseline (Fig. 5A). On protein level, as assessed by immunostaining, we observed a similar trend of upregulation of aSMA and FN1 in hPCLS stored in both solutions after FC treatment (Fig. 5B and Suppl. Figure 5A). The increase in FN1 upon FC showed a significant correlation between samples treated at day 0 and samples treated after cold storage, suggesting a similar fibrotic response before and after cold storage (Suppl. Figure 5B). In conclusion, TiProtec (-) and TiProtec can effectively maintain the fibrotic response capacity of hPCLS after 14 days of cold storage.

## Discussion

Chronic lung diseases remain a leading cause of morbidity and mortality worldwide [1]. hPCLS are a versatile model for studying disease-associated mechanisms [2, 3], pre-clinical drug testing [5–7], and toxicology assessments [8–10]. hPCLS maintain the intact 3D lung architecture and contain all the resident cell types like airway epithelial cells, type I and type II alveolar cells, fibroblasts, immune cells (e.g. alveolar macrophages), and smooth muscle cells [11–13]. However, one of the main limitations that researchers face while working with hPCLS is the limited tissue availability. This limitation, compounded by the low throughput slice generation and lack of adequate storage options, forces researchers to use freshly generated hPCLS immediately and discard any remaining tissue, since it not possible to keep them for later experiments. Additionally, researchers not working at major transplant or thoracic surgery centers do not have access to fresh lung tissue. Therefore, having the possibility to store, ship, and use hPCLS on-demand is of great importance for the community.

Here, we studied the potential for long-term cold storage of hPCLS in DMEM/F-12, TiProtec, and TiProtec without iron chelators. After 7 days, viability and metabolic activity were reduced and a significant reduction in immune cells such as macrophages as well as a significant decrease in specific epithelial populations such as alveolar type I and type II cells [31] just after 7 days of cold storage were observed with DMEM/F-12, suggesting that long-term storage in this solution is not feasible. TiProtec is a derivative of the organ preservation solution Custodiol-N [59–62], that is optimized for blood vessels, cells, and tissue storage [47, 63]. It was originally optimized for the cold storage of cells and vascular grafts [44, 47, 63, 64]. Since then, it has been used for cold storage of human HepaRG liver spheroids, human hepatocytes, liver-on-chip organ models, endothelialized gas-exchange membranes, and muscle tissue [48, 64–67]. Its tissue preserving properties are attributed to the presence of the amino acids alanine and glycine, which prevent hypoxic cell injury [68–70]; a risk that is particularly high for hPCLS due to their thickness (500 μm) [71]. The inhibition of the formation of nonspecific, hypoxia-induced plasma membrane pores by glycine (and to a lesser extent by alanine)– and subsequent inhibition of sodium and calcium influx into the hypoxic cells– has been shown to be the molecular mechanism of this protection [68, 70, 72, 73]. In addition, TiProtec includes α-ketoglutarate, shown to act as an antioxidant [74] and as an intermediate in the tricarboxylic acid cycle supporting ATP production and stabilizing cellular membranes in conjunction with tryptophan [75]. High aspartate concentrations ensure the energy supply through the tricarboxylic acid cycle, *N*-acetylhistidine maintains pH balance at a slightly acidic, protective pH, and sucrose prevents osmotic cell swelling [47, 76, 77]. Low calcium levels offer protection against various types of cellular injuries [78, 79]. The iron chelators LK 614, a small lipophilic hydroxamic acid derivative, and deferoxamine, a large, hydrophilic hexadentate chelator of Fe^3+^, were incorporated into TiProtec to inhibit iron-dependent reactive oxygen species (ROS) formation, the major pathway of hypothermic injury in many cell types [41, 43, 64, 80]. Hypothermia has been shown to trigger the release of tightly bound iron ions into the „free“ form, i.e. to lead to an increase in the cellular chelatable, redox-active iron pool [43, 80, 81]. These iron ions then give rise to the formation of highly reactive oxygen species and subsequent mitochondrial permeability transition [41, 43, 80, 81]. This iron-dependent, ROS-mediated cold-induced injury occurred in many cell types such as human and rat hepatocytes, diverse macro- and microvascular endothelial cells, rat renal tubules, proximal tubular cells and, of note, the lung tumor cell line A549 [40, 41, 43, 46, 47, 81, 82]. It proved to be inhibitable by the iron chelators deferoxamine, 2,2´-dipyridyl, and 1,10-phenanthroline as well as by the combination of deferoxamine and LK 614 [40, 41, 43, 82]. Our findings demonstrate that TiProtec outperforms DMEM/F-12 in preserving hPCLS during long-term cold storage across all analyzed endpoints. Long-term cold storage for up to 14 days was possible without significant reductions in viability or metabolic activity (Fig. 1A, C*)*, while storage for up to 28 days in TiProtec showed minimal changes regarding viability and histological changes. Interestingly, most of our results are consistent with findings from Tigges et al. [31], which points to no species-specific differences in viability and metabolic activity between human and rat PCLS.

Given the finding that iron-dependent injury is the major cold-induced injury in many cell types including the lung tumor cell line A549, it is surprising that the addition of the iron chelators LK 614 and deferoxamine did not only not improve cellular metabolic function after cold storage of hPCLS– a finding that would point to a relative low sensitivity of hPCLS, similar to rat PCLS [31, 43, 46], to iron-dependent hypothermic injury (likely due to a certain degree of hypoxia in the thick slices, limiting the formation of reactive oxygen species); the iron chelators even enhanced loss of metabolic activity following prolonged cold storage periods (Fig. 1C). Possibly, this unfavorable effect of the chelators in our current model is related to unwanted cellular iron depletion. Iron depletion by deferoxamine has been described to lead to apoptosis during incubation at 37 °C [83–85]. It appears worthwhile to assess whether rewarming in a chelator-free solution or cell culture medium instead of TiProtec would yield superior metabolic recovery. This is of particular interest as transcriptomics analysis and the evaluation of senescence induction suggest that the addition of iron chelators is beneficial for the cold storage of hPCLS. However, the major effect of TiProtec and Tiprotec (-) on viability and metabolic activity in our model is thus likely due to the protective effects of the amino acids glycine and alanine against hypoxic injury/energy deficiency injury.

This is the first study addressing the transcriptional changes associated with cold storage of hPCLS and providing a reference gene expression dataset that can be used for future studies. A cell type signature enrichment analysis revealed the preservation of most cellular compartments upon cold storage on the transcriptional level. In addition, transcriptional signatures revealed downregulation of pathways related to inflammation as well as, cell death, and oxidative stress, which are detrimental for cellular function after cold storage because they can lead to cellular senescence and lower regeneration capacity in the explanted tissues [31, 86, 87]. This confirms that TiProtec confers its tissue protective functions via regulation of these stress response pathways in lung tissue. Consistently, TiProtec and TiProtec (-) also shielded hPCLS from senescence induced by cold storage. These observations suggest that lung slices may exhibit greater resistance to cold storage-induced senescence, a phenomenon previously described in organs stored in such conditions [86, 87]. Future studies will need to focus on cell-type specific regulation of stress pathways and their upstream regulators upon cold storage. The downregulation of pathways related to inflammation is likely to be– in part– the result of lower injury in TiProtec and TiProtec (-) (Fig. 1A, C) and thus less activation of innate immune cells by released danger-associated molecular patterns (DAMPs). In addition, the amino acid glycine is a potent anti-inflammatory agent [73, 88, 89]. Glycine is known to activate glycine receptors which are glycine-gated chloride channels. These are expressed by macrophages [88], including alveolar macrophages, and other immune cells. Activation of the glycine-gated chloride channels leads to chloride influx and hyperpolarization that decreases the open probability of calcium channels and thus macrophage/immune cell activation. Thus, both, the cytoprotective effect of glycine (action on non-specific, hypoxia-induced membrane pores) and the inhibitory effect of glycine on macrophage activation likely contribute to the downregulation of pathways related to inflammation observed here. The maintenance of low inflammation and senescence levels during cold storage in TiProtec and TiProtec (-) are a great advantage since this is of high importance for the usage of these hPCLS in disease-relevant translational research. Moreover, as the anti-inflammatory effect requires the presence of the ligand glycine, the anti-inflammatory effect is observed during and immediately after cold storage but is unlikely to compromise the subsequent use of the cold-stored hPCLS in (inflammatory) disease models after the hPCLS have been returned to their usual culture medium.

The downregulation of the hypoxia response in the iron chelator-containing solution (Fig. 3B) is unexpected as deferoxamine is well known to activate hypoxia-inducible factor-1a (HIF-1a) [90, 91]. However, one must consider that samples for this analysis were taken directly after cold storage without any rewarming. Processes like the translocation of HIF-1a to the nucleus might need rewarming (e.g. for reconstitution of disintegrated microtubules), similarly, the transcriptional response to HIF-1a might require more physiological temperatures. Additional assessment of these responses after rewarming would therefore be an important next step.

Finally, to assess the functional response of the cold-stored hPCLS, we exposed them to a fibrotic cocktail, previously used to study the pathobiology of IPF [2, 23, 49]. hPCLS cold-stored in TiProtec preserved their responsiveness to fibrotic stimuli when compared to freshly cut hPCLS. This highlights the applicability of this storage technique for translational lung research applications as well as increasing collaborative potential since this method allows the transfer of hPCLS with very simple transportation requirements [32]. Additionally, the evaluation of other disease-relevant stimuli [92] would determine the broad applicability of this cold storage method. Beyond its implications for basic research, cold storage of hPCLS in TiProtec holds significant potential for advancing therapeutic applications. By enabling the preservation of hPCLS for at least up to 14 days, this approach can support drug discovery and screening in chronic lung disease models, providing a more physiologically relevant platform compared to traditional 2D cell cultures. Moreover, TiProtec facilitates the simultaneous use of hPCLS from different patients through a possible stockpiling strategy, which could allow for high-throughput therapeutic testing. This capability is particularly advantageous for investigating inter-patient variability and tailoring treatments to specific disease phenotypes or patient populations. By offering a scalable solution for preserving and utilizing patient-derived lung tissue, TiProtec could accelerate the development of personalized therapies and enhance the translational potential of preclinical lung disease models. However, this study is limited by the fact that lung tissue derived from different disease identities and sources was not used, as our experiments were restricted to tissues from patients undergoing cancer resection surgery [93]. Furthermore, changes in cellular composition were assessed only at the transcriptomic level and not in a cell-type specific manner; future studies should incorporate cell-type specific analysis on the protein level. Additionally, further research is needed to characterize the differential susceptibility to cold storage or cryopreservation of slices with different compositions and a side-to-side comparison to other long-term storage options including cryopreservation would be desirable. Although cryopreservation has the inherent danger of damaging cells, it might allow for longer storage time and Patel et al. report that the cryopreserved hPCLS retain viability and immune responsiveness for up to 28 days [32]. Despite these limitations, our findings provide a foundation for further exploration of this storage method and its applicability to studying disease-specific mechanisms.

## Conclusions

In conclusion, our study presents a novel method for long-term cold storage of hPCLS that preserves their viability, metabolic activity, transcriptional profile, and cellular composition for up to 14 days. From the 3 solutions tested, TiProtec performed the best and therefore, we would recommend the usage of this solution for future studies. Finally, our study demonstrated that cold-stored hPCLS can be used for on-demand mechanistic studies relevant for respiratory research.

## Electronic supplementary material

Below is the link to the electronic supplementary material.

Supplementary Material 1

Supplementary Material 2

Supplementary Material 3

Supplementary Material 4

Supplementary Material 5

Supplementary Material 6

## Data Availability

No datasets were generated or analysed during the current study.
