# Supplementary figures and images for: Cold storage of human precision-cut lung slices in TiProtec preserves cellular composition and transcriptional responses and enables on-demand mechanistic studies
Source: Respir Res. 2025 Feb 17;26:57. doi: 10.1186/s12931-025-03132-w (PMC11834602; doi:10.1186/s12931-025-03132-w)

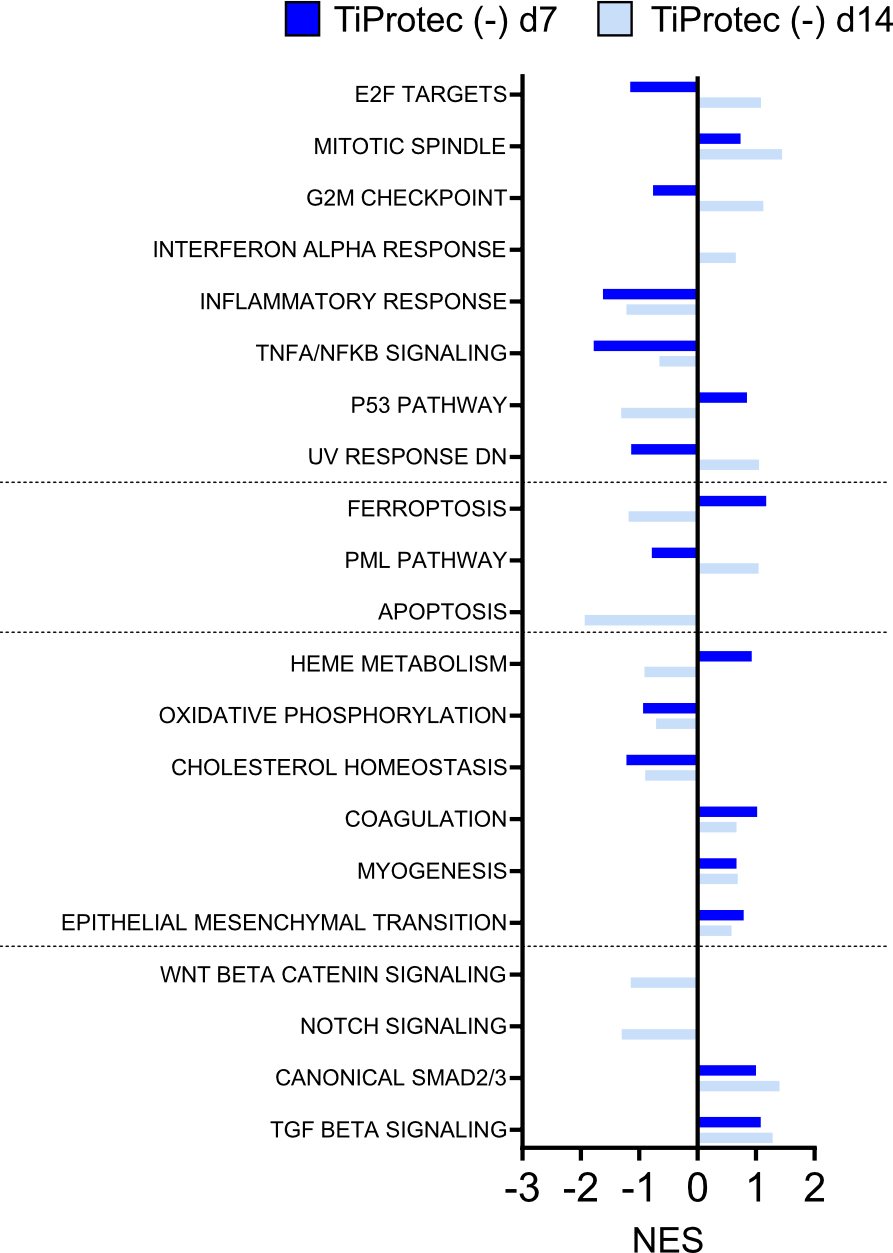

Supplement: Supplementary file 1 — Supplementary Material 1 [file 12931_2025_3132_MOESM1_ESM.tiff]

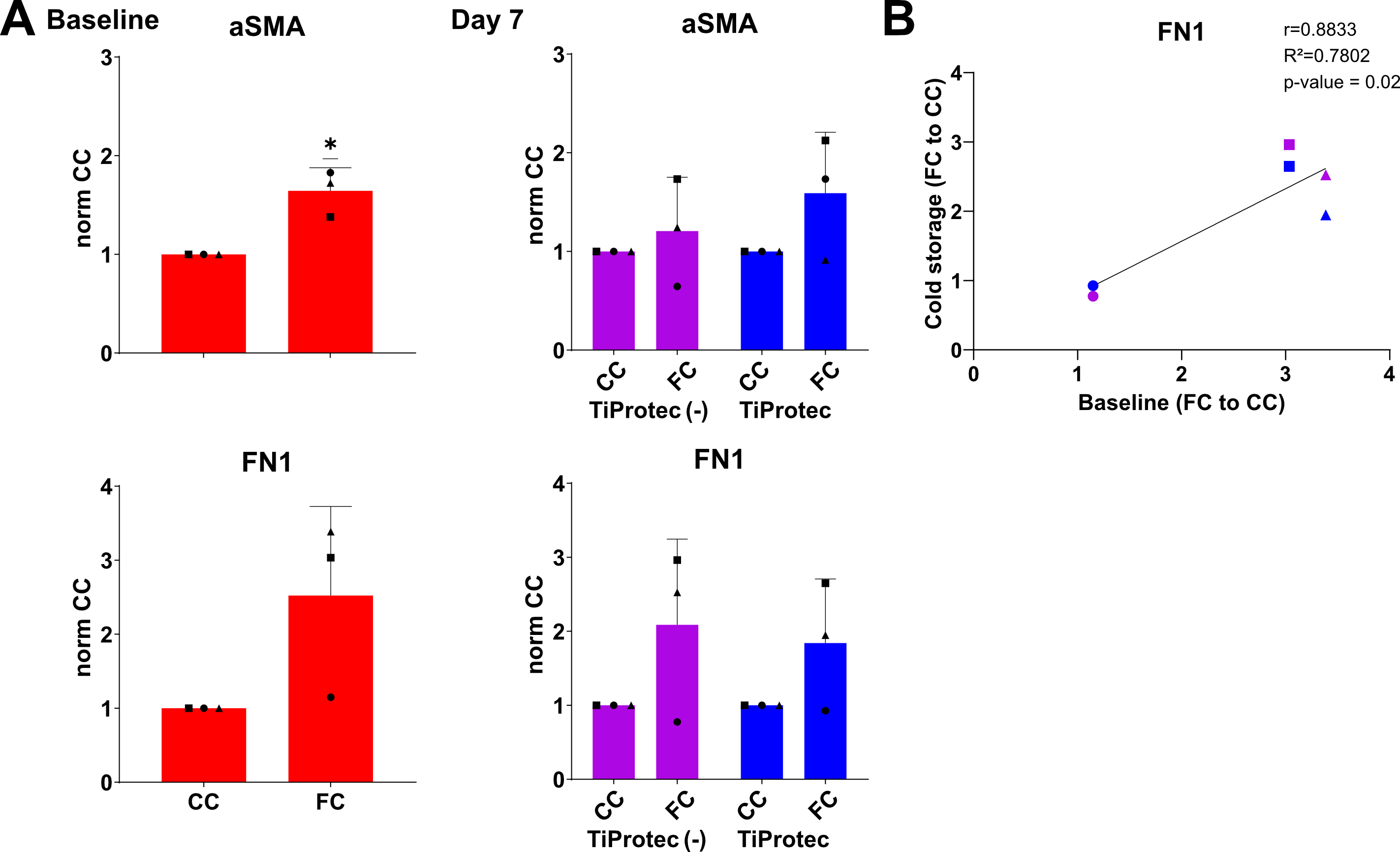

Supplement: Supplementary file 2 — Supplementary Material 2 [file 12931_2025_3132_MOESM2_ESM.tiff]

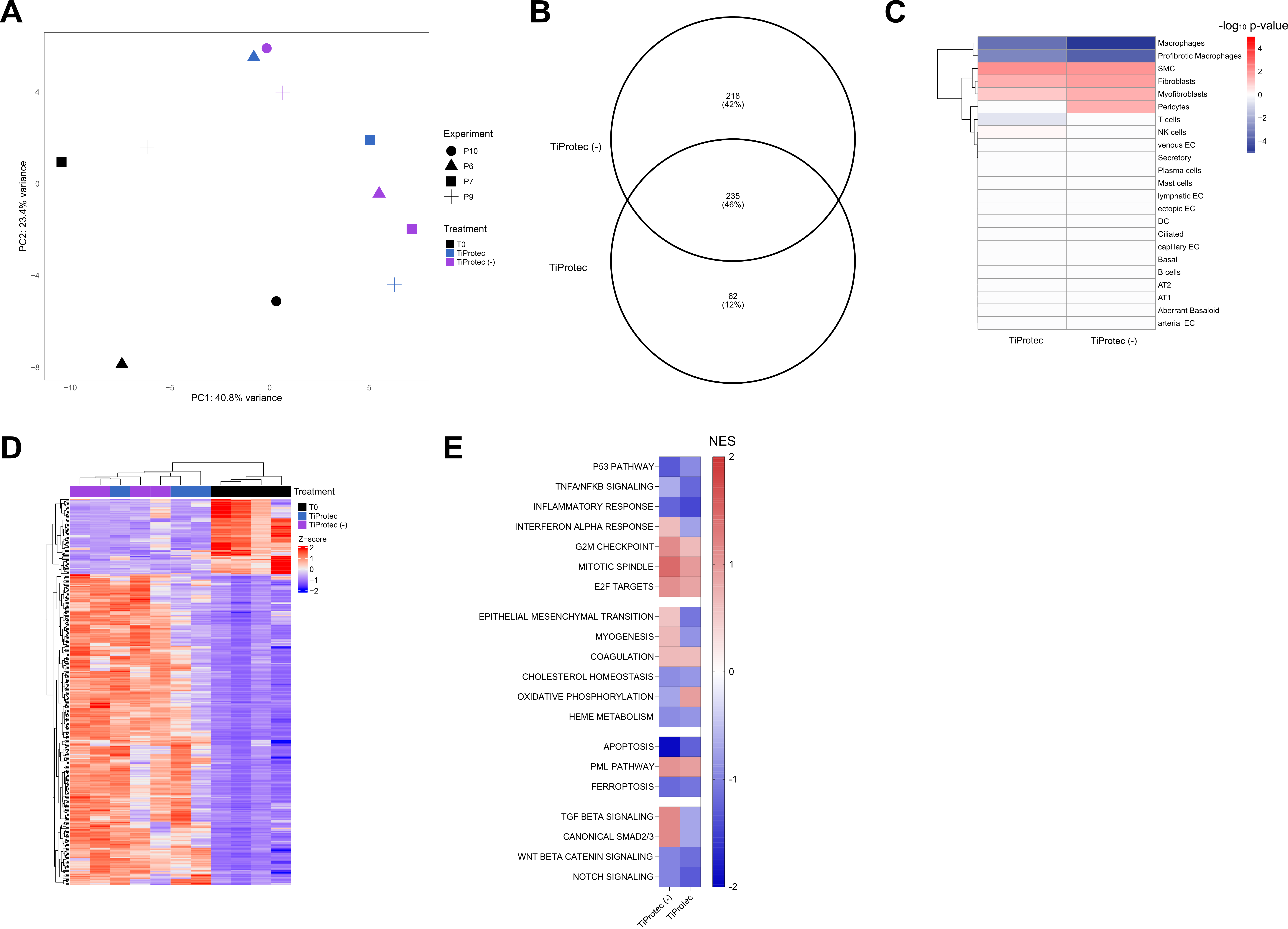

Supplement: Supplementary file 3 — Supplementary Material 3 [file 12931_2025_3132_MOESM3_ESM.tiff]

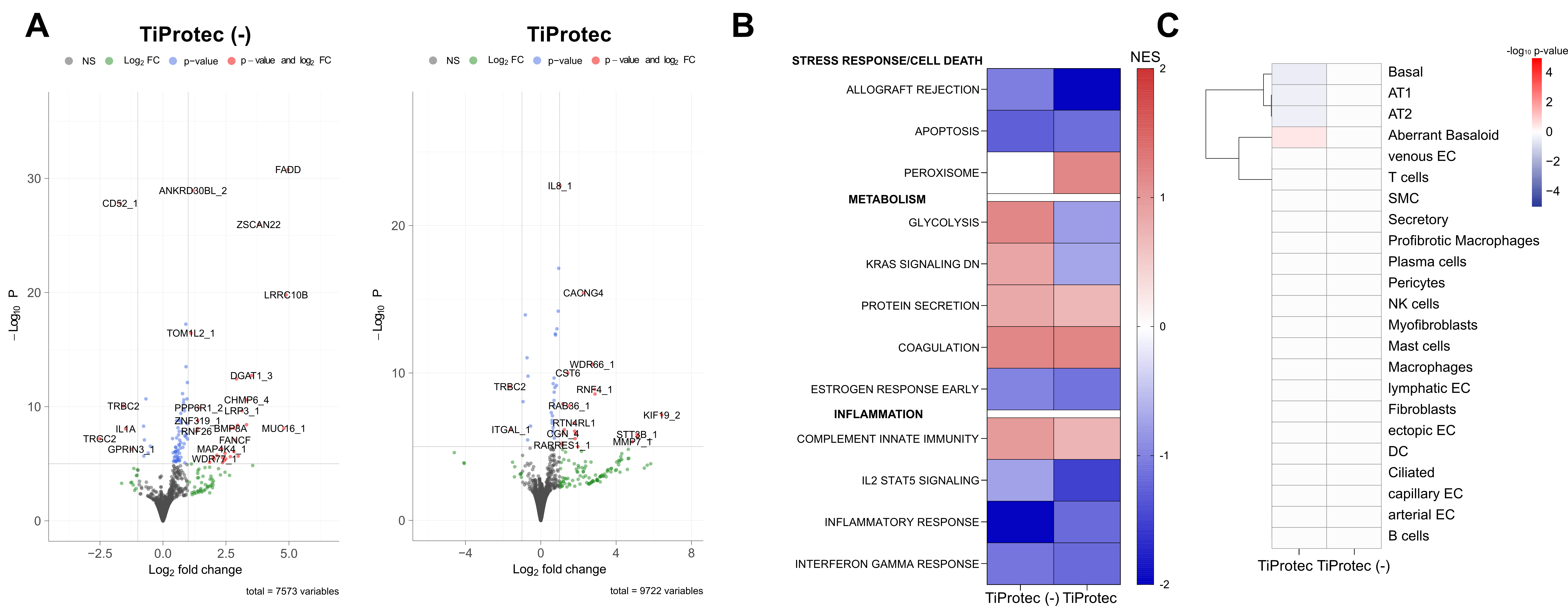

Supplement: Supplementary file 4 — Supplementary Material 4 [file 12931_2025_3132_MOESM4_ESM.tiff]

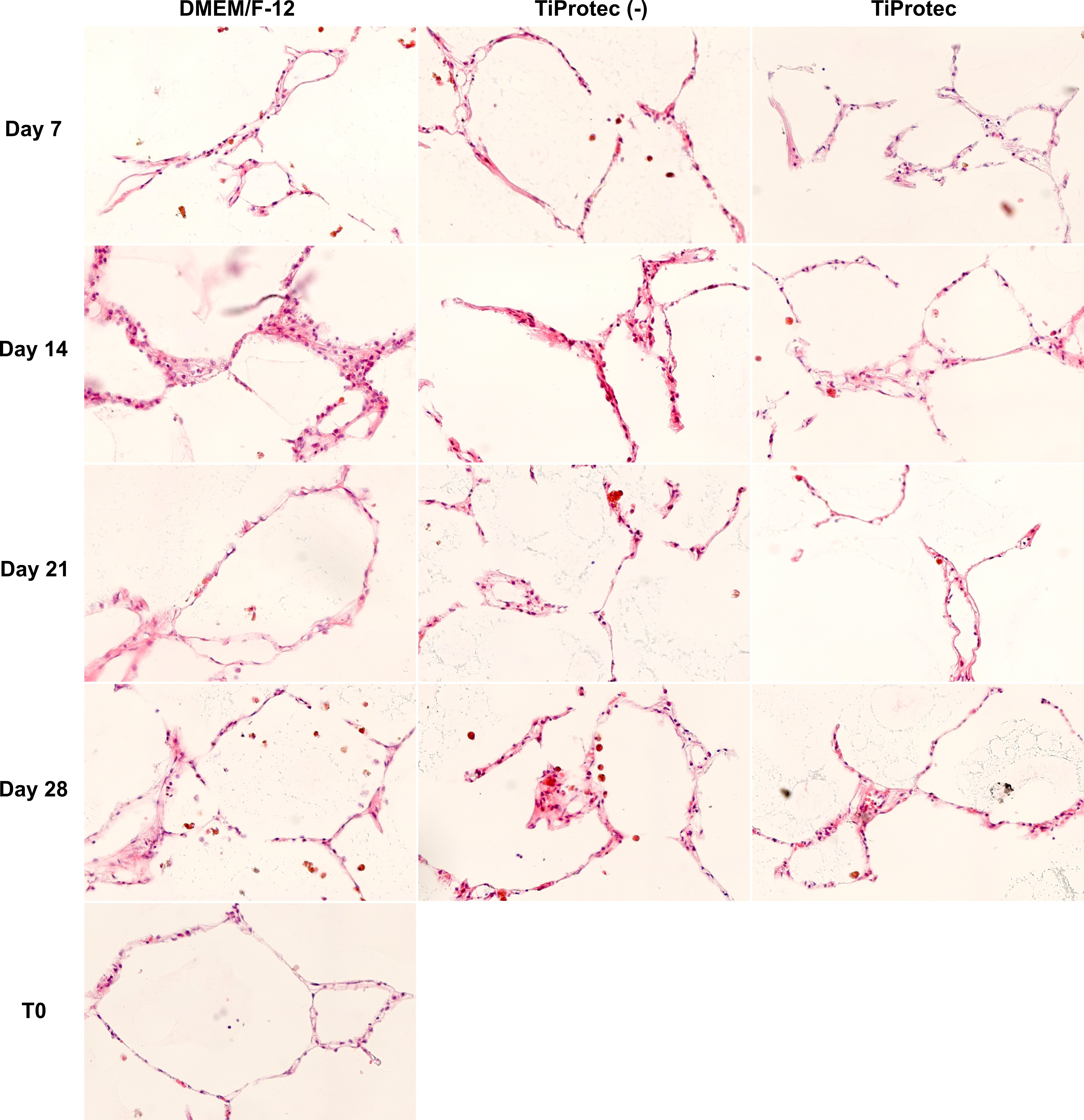

Supplement: Supplementary file 5 — Supplementary Material 5 [file 12931_2025_3132_MOESM5_ESM.tiff]
