# Supplementary material for: Cold storage of human precision-cut lung slices in TiProtec preserves cellular composition and transcriptional responses and enables on-demand mechanistic studies
Source: Respir Res. 2025 Feb 17;26:57. doi: 10.1186/s12931-025-03132-w (PMC11834602; doi:10.1186/s12931-025-03132-w)
Supplement: Supplementary file 6 — Supplementary Material 6 [file 12931_2025_3132_MOESM6_ESM.docx]

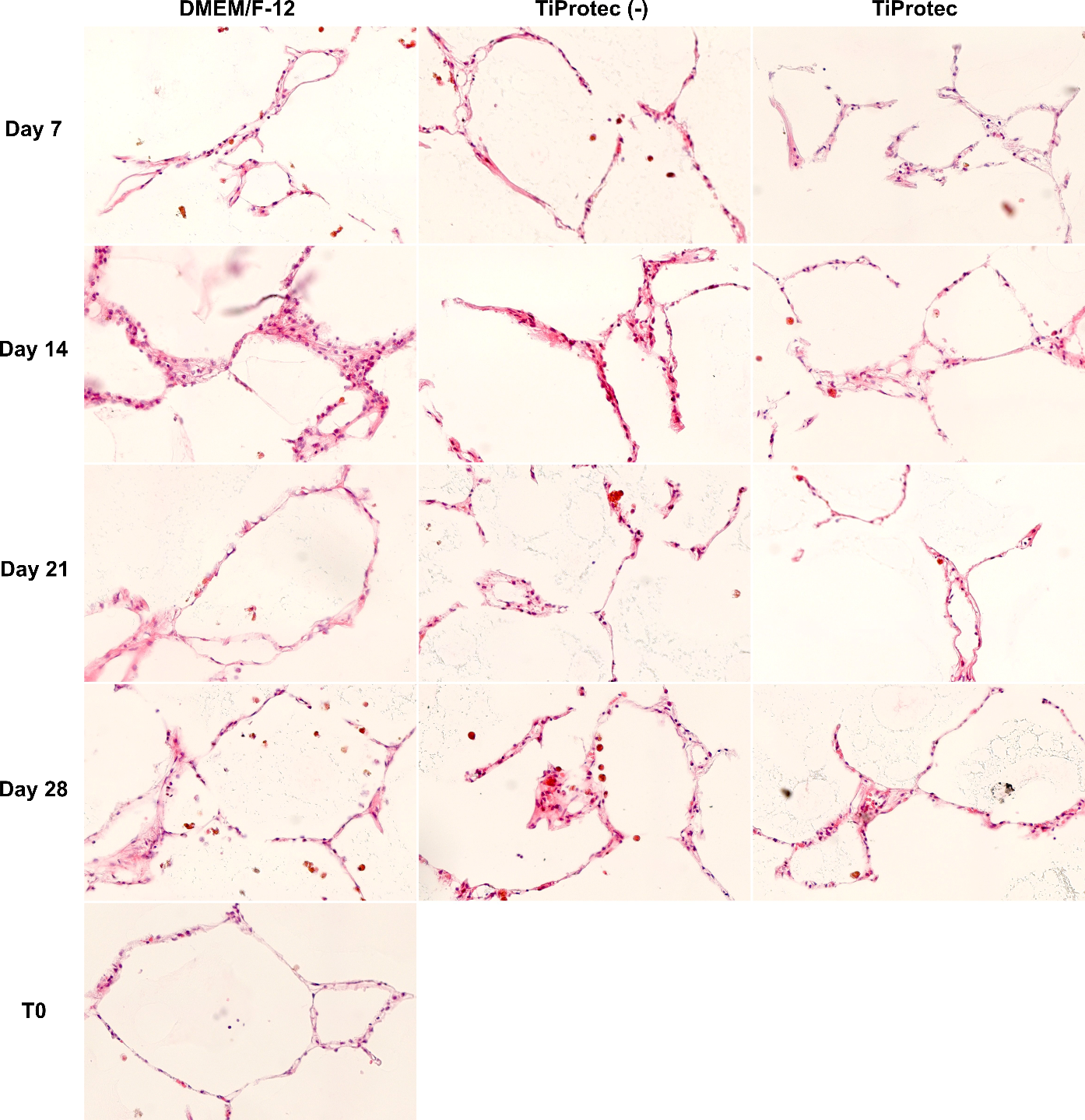


**Supplementary Figure 1. Histological characterization at different time points after cold storage of hPCLS.** Representative images of H&E staining of hPCLS fixed at 0, 7, 14, 21, and 28 days after slicing and cold storage in DMEM/F-12, TiProtec (-), or TiProtec.


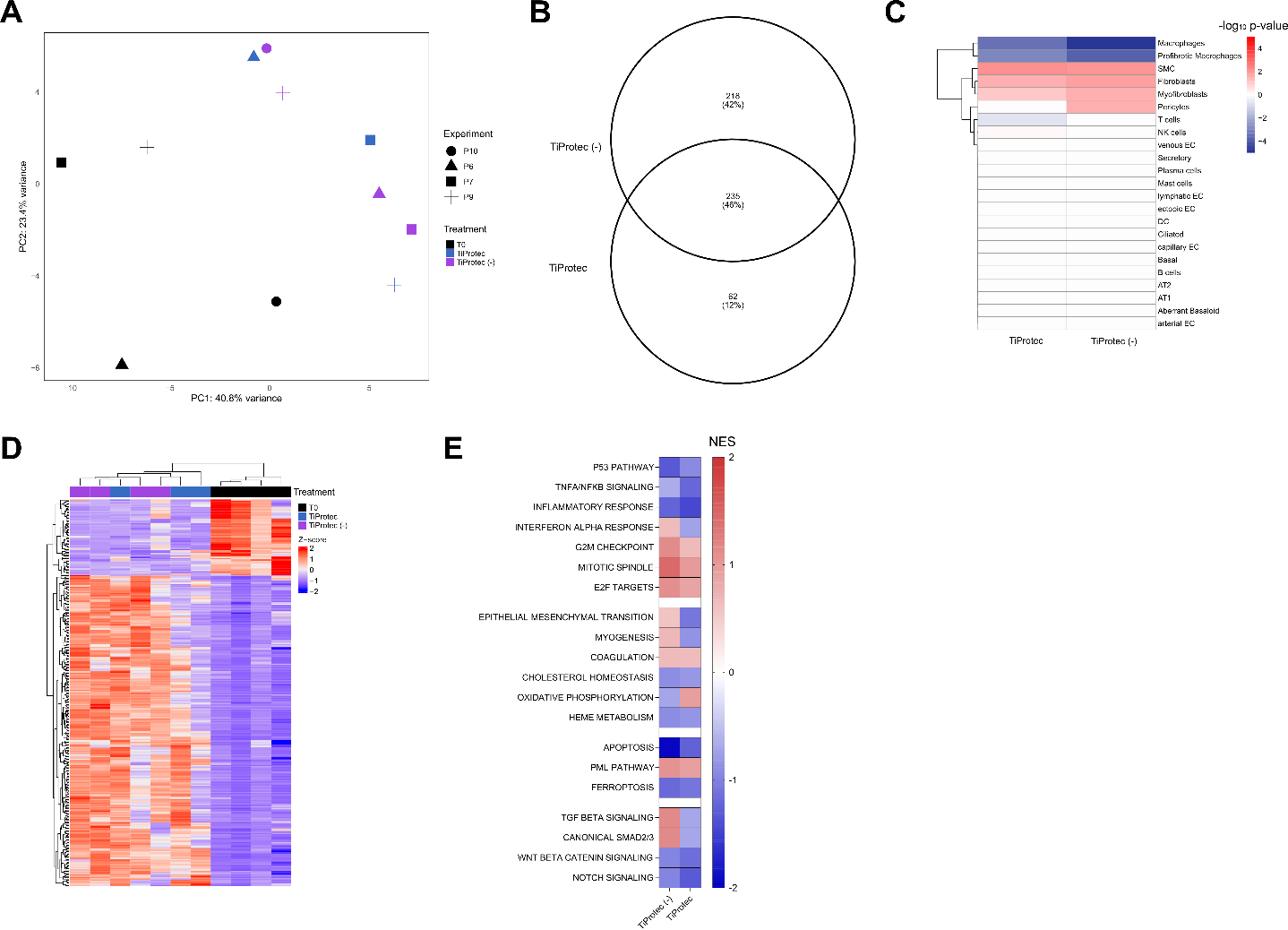
**Supplementary figure 2. Transcriptional changes induced by TiProtec (-) and TiProtec after 14 days of cold storage in comparison to T0 control.** A) Principal component analysis for freshly cut hPCLS (T0) and after 14 days of cold storage in TiProtec (-) or TiProtec. Shapes show different three (TiProtec) or four (TiProtec (-) biological replicates and colors indicate the cold storage solution. B) Venn diagram of DEG after 14 days of cold storage when compared to T0 baseline control (LFC > 0). C) Deconvolution analysis for gene signatures of main cellular compartments in hPCLS based on DEG displayed in B. D) Heatmap of DEG after 14 days of cold storage in TiProtec (-) or TiProtec when compared to T0 baseline control (LFC > 1). E) Deregulated pathways in hPCLS after 14 days of cold storage in TiProtec or TiProtec (-) based on DEG from D.


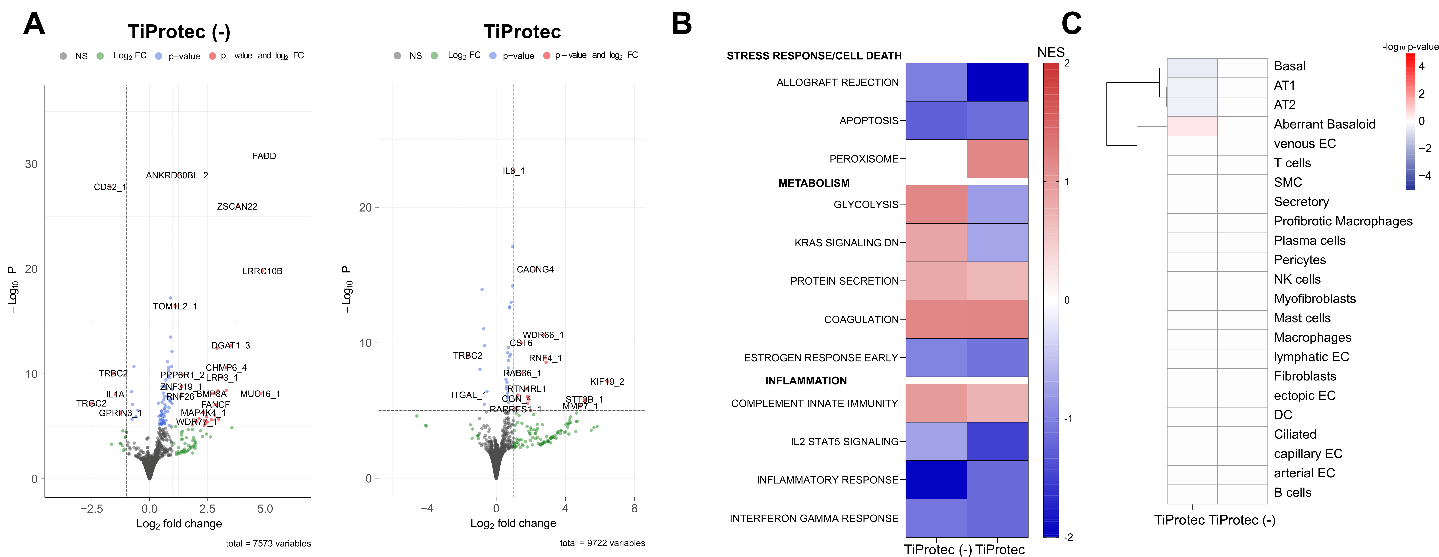
**Supplementary figure 3. Transcriptional changes induced by TiProtec and TiProtec (-) after 14 days of cold storage in comparison to day 7.** A) Volcano plot of differentially expressed genes (DEG) after 14 days of cold storage in TiProtec or TiProtec (-) when compared to hPCLS stored for 7 days. Data is from hPLCS obtained from at least three different biological replicates. Significant DEG are highlighted in red and corresponding gene names are shown. B) Gene set enrichment analysis of deregulated pathways in hPCLS after 14 days of cold storage based on DEG from A. C) Deconvolution of cellular compartment significantly changed in hPCLS after 14 days of cold storage with TiProtec or TiProtec (-) when compared to day 7.


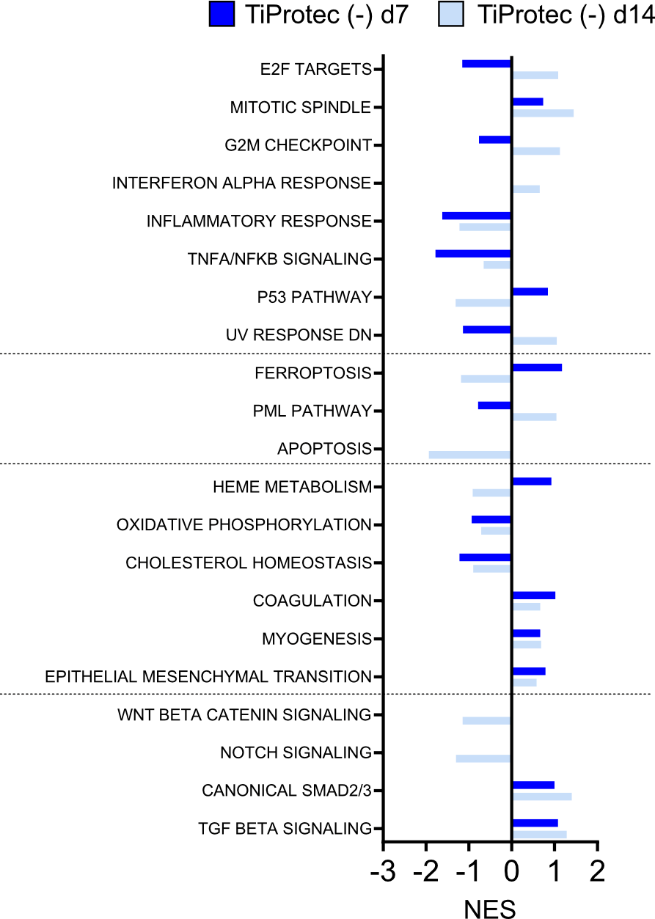


**Supplementary figure 4. Transcriptional changes induced by TiProtec (-) after 7 and 14 days of cold storage in comparison to T0 controls.** Deregulated pathways in hPCLS after 7 and 14 days of cold storage in TiProtec (+) based on DEG in comparison to T0 control (LFC > 1).


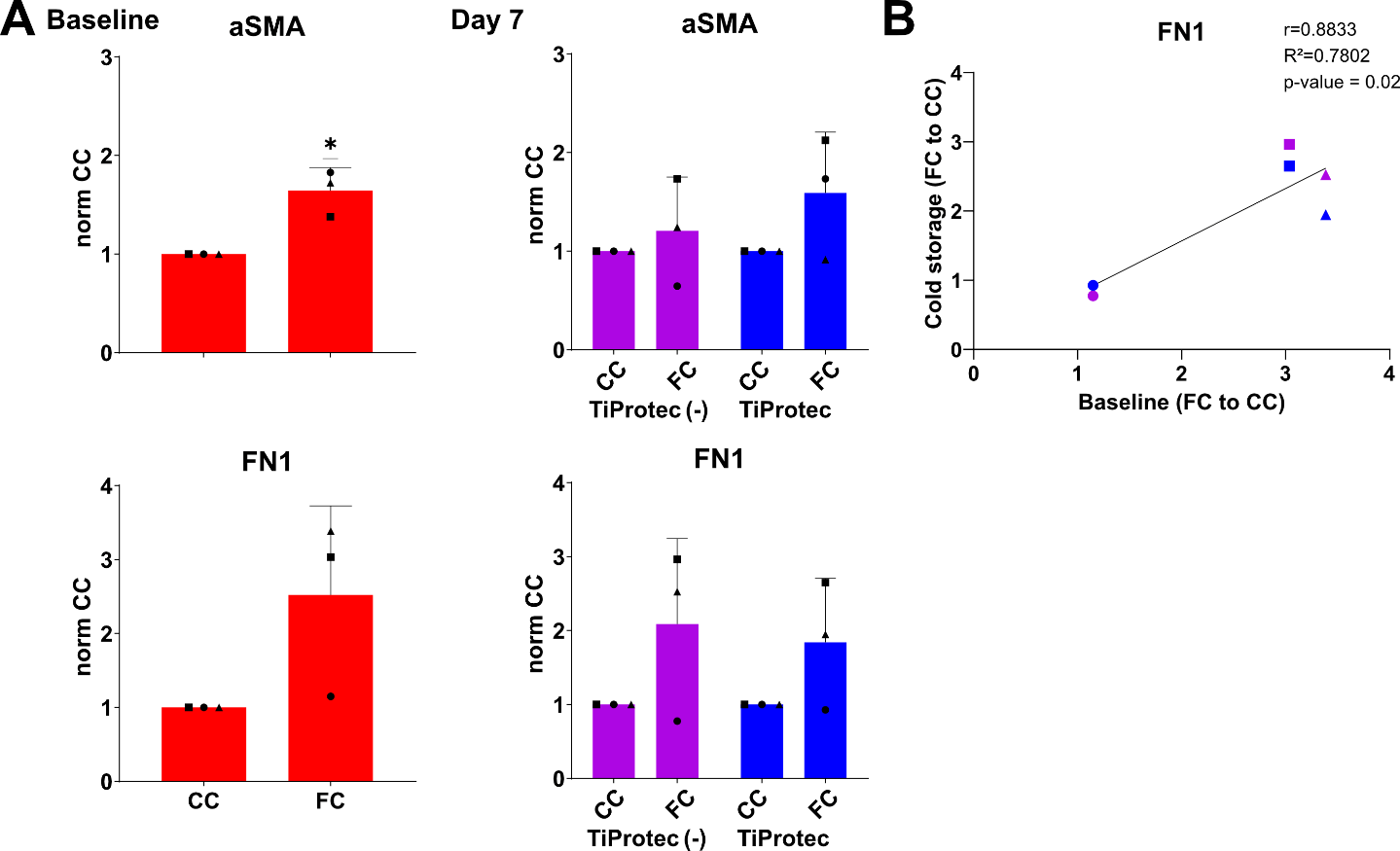


**Supplementary Figure 5. Quantification of immunostaining for fibrosis-related proteins in hPCLS cold stored for 7 days and stimulated with a fibrotic cocktail.** At baseline or after 7 days of cold storage in TiProtec or TiProtec (-), hPCLS were treated with CC or FC. Immunostaining was used to determine the expression of the fibrosis-related proteins: aSMA and FN1. A. Mean fluorescence intensity for each protein was normalized to total cell number based on DAPI signal. Bar represent mean + SD and single points with different shapes represent different biological replicates (N=3). * p<0.05 after one-sample t-test. B. Pearson correlation analysis of fold change in protein expression at baseline and after cold storage. Single points with different shapes represent different biological replicates (N=3) and color indicate the cold storage solution. P-value < 0.5 were considered significant.

|  | DMEM/F-12 |
| --- | --- |
| CaCl_2_ • 2 H_2_O | 0.1545 |
| CuSO_4_ • 5 H_2_O | 0.0000013 |
| Fe(NO_3_)_3_ • 9 H_2_O | 0.00005 |
| FeSO_~~4~~_ • 7 H_2_O | 0.000417 |
| MgCl_2_ • 6 H_2_O | 0.0612 |
| MgSO_4_ | 0.04884 |
| KCL | 0.3118 |
| NaHCO_3_ | 1.2 |
| NaCl | 6.996 |
| Na_2_HPO_4_ | 0.07102 |
| NaH_2_PO_4_ | 0.0543 |
| ZnSO_4_ • 7 H_2_O | 0.000432 |
| L-Alanine | 0.00445 |
| L-Arginine • HCl | 0.1475 |
| L-Asparagine • H_2_O | 0.0075 |
| L-Aspartic Acid | 0.00665 |
| L-Cystine • 2 HCl | 0.03129 |
| L-Cysteine • HCl • H_2_O | 0.01756 |
| L-Glutamic Acid | 0.00735 |
| Glycine | 0.01875 |
| L-Histidine • HCl • H_2_O | 0.03148 |
| L-Isoleucine | 0.05447 |
| L-Leucine | 0.05905 |
| L-Lysine • HCl | 0.09125 |
| L-Methionine | 0.01724 |
| L-Phenylalanine | 0.03548 |
| L-Proline | 0.01725 |
| L-Serine | 0.02625 |
| L-Threonine | 0.05345 |
| L-Tryptophan | 0.00902 |
| L-Tyrosine • 2 Na • H_2_O | 0.05579 |
| L-Valine | 0.05285 |
| D-Biotin | 0.0000035 |
| Choline Chloride | 0.00898 |
| Folic Acid | 0.00266 |
| myo-Inositol | 0.0126 |
| Niacinamide | 0.00202 |
| D-Pantothenic Acid • ½ Ca | 0.00224 |
| Pyridoxine • HCl | 0.002031 |
| Riboflavin | 0.000219 |
| Thiamine • HCl | 0.00217 |
| Vitamin B12 | 0.00068 |
| D-Glucose | 3.15 |
| HEPES | 3.5745 |
| Hypoxanthine | 0.00244 |
| Linoleic Acid | 0.000042 |
| Putrescine • 2 HCl | 0.000081 |
| Pyruvic Acid • Na | 0.055 |
| DL-Thioctic Acid | 0.000105 |
| Thymidine | 0.000365 |

**Supplementary Table 1. Complete DMEM/F-12 (Product ID: 6434-500mL) composition.** Concentrations are given in g/L.
